# A novel multi-strategy ameliorated quasi-oppositional chaotic tunicate swarm algorithm for global optimization and constrained engineering applications

**DOI:** 10.1016/j.heliyon.2024.e30757

**Published:** 2024-05-09

**Authors:** Vanisree Chandran, Prabhujit Mohapatra

**Affiliations:** Department of Mathematics, Vellore Institute of Technology, Vellore, 632014, Tamil Nadu, India

**Keywords:** Meta-heuristic algorithms, Tunicate swarm algorithm (TSA), Chaotic maps, Quasi-oppositional based learning (QOBL), Engineering design problems

## Abstract

Over the last few decades, a number of prominent meta-heuristic algorithms have been put forth to address complex optimization problems. However, there is a critical need to enhance these existing meta-heuristics by employing a variety of evolutionary techniques to tackle the emerging challenges in engineering applications. As a result, this study attempts to boost the efficiency of the recently introduced bio-inspired algorithm, the Tunicate Swarm Algorithm (TSA), which is motivated by the foraging and swarming behaviour of bioluminescent tunicates residing in the deep sea. Like other algorithms, the TSA has certain limitations, including getting trapped in the local optimal values and a lack of exploration ability, resulting in premature convergence when dealing with highly challenging optimization problems. To overcome these shortcomings, a novel multi-strategy ameliorated TSA, termed the Quasi-Oppositional Chaotic TSA (QOCTSA), has been proposed as an enhanced variant of TSA. This enhanced method contributes the simultaneous incorporation of the Quasi-Oppositional Based Learning (QOBL) and Chaotic Local Search (CLS) mechanisms to effectively balance exploration and exploitation. The implementation of QOBL improves convergence accuracy and exploration rate, while the inclusion of a CLS strategy with ten chaotic maps improves exploitation by enhancing local search ability around the most prospective regions. Thus, the QOCTSA significantly enhances convergence accuracy while maintaining TSA diversification. The experimentations are conducted on a set of thirty-three diverse functions: CEC2005 and CEC2019 test functions, as well as several real-world engineering problems. The statistical and graphical outcomes indicate that QOCTSA is superior to TSA and exhibits a faster rate of convergence. Furthermore, the statistical tests, specifically the Wilcoxon rank-sum test and *t*-test, reveal that the QOCTSA method outperforms the other competing algorithms in the domain of real-world engineering design problems.

## 1 Introduction

### 1.1 Motivation and incitement

Optimization is the systematic procedure of identifying the best optimal solution for a specific problem within a given set of constraints. Based on these characteristics, optimization algorithms can be categorized into two main groups, namely deterministic and stochastic approaches [1], [2]. A deterministic approach consistently generates the same values for a given set of inputs. These are gradient-restricted techniques that systematically approach the best optimal value. On the contrary, stochastic approaches are gradient-free methods that employ random steps to achieve the optimal value. This optimization approach cannot be repeated under any circumstances. In other words, the stochastic approach generates diverse solutions because of random fluctuations. Stochastic approaches are further categorized into two kinds, namely heuristic and meta-heuristic algorithms [3]. In optimization, a Heuristic Algorithm (HA) is a process of finding a close-to-optimal solution. On the other hand, they generate feasible solutions in a specified number of steps, indicating problem dependence [4], [5]. In general, a heuristic is a procedure that employs the trial-and-error method to find solutions in an appropriate computational time and space. However, due to their greedy nature, heuristic algorithms fail to ensure the global optimal value, getting trapped in the local optimum and exhibiting rapid convergence. Meta-heuristic algorithms (MHAs) have been developed because of the shortcomings of heuristic algorithms. Each MHA employs its own unique strategies to direct the search process with the goal of effectively exploring the search region to locate close-to-optimal solutions. MHA encompasses a variety of techniques, from simple local exploration to intricate learning procedures. These algorithms are considered problem-independent because they do not take advantage of any problem specifics. Furthermore, they are non-greedy in nature, which facilitates them to extensively explore the search region, perhaps leading to a superior solution that occasionally coincides with global optima. Therefore, MHAs are capable of identifying the superior outcomes to all optimization problems without becoming stuck in local optimal traps [6]. These efficient and robust algorithms are applied to solve a variety of problems, including path planning [7], feature selection [8], wireless sensor network [9], image processing [10], and neural networks [11].

### 1.2 Literature review

**Figure 1:**
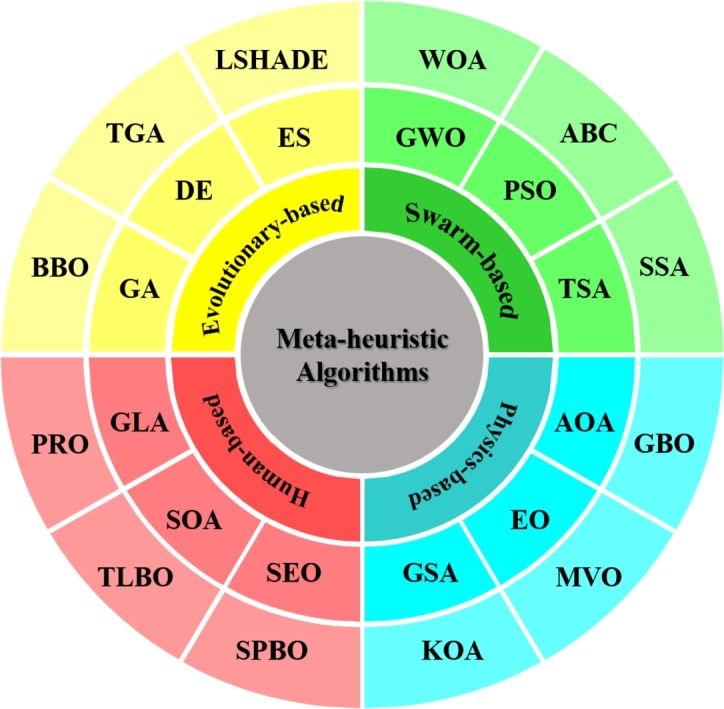
Classification of MHAs.

Meta-heuristic algorithms mainly involve two key attributes: exploration and exploitation. Exploration refers to an algorithm's ability to globally investigate diverse sections of the search region. This prevents local optimal entrapment and swifts away from the stagnation of the local optimal regions. Exploitation refers to the ability to locally explore for possible solutions in all promising regions to enhance the solution's accuracy. A satisfactory performance is obtained by balancing these two key attributes to achieve optimal or close-to-optimal solutions. Inspired by this, MHAs are typically classified into evolutionary-based, swarm-based, physics-based, and human-based algorithms as shown in Fig. 1. Evolutionary-based algorithms imitate the theories of biological evolution of organisms in nature; swarm-based algorithms simulate the social and collective behaviour of swarms, including colonies of ants, flocks of birds, herds of animals, and so on; physics-based algorithms mimic the fundamental laws of physics in the universe; and human-based algorithms stimulate the behavioural patterns of humans in the world [12]. Some of the well-known and widely employed nature-inspired MHAs are Differential Evolution (DE) [13], Particle Swarm Optimization (PSO) [14], Genetic Algorithm (GA) [15], Ant Colony Optimization (ACO) algorithm [16], Artificial Bee Colony (ABC) algorithm [17], and Grey Wolf Optimization (GWO) algorithm [18]. In recent years, researchers have become more interested in these algorithms due to their high-quality solutions to optimization problems in the fields of engineering and science. In addition to this, several recently introduced nature-inspired MHAs are Success History-based Adaptive Differential Evolution with Linear Population Size Reduction (LSHADE) [19], Evolutionary Strategy (ES) [20], Bio-geography Based Optimization (BBO) [21], Tree Growth Algorithm (TGA) [22], Tunicate Swarm Algorithm (TSA) [23], Marine Predators Algorithm (MPA) [24], White Shark Optimizer (WSO) [25], Aquilla Optimizer (AO) [26], Seagull Optimization Algorithm (SOA) [27], Whale Optimization Algorithm (WOA) [28], Coati Optimization Algorithm (COA) [29], Farmland Fertility Algorithm (FFA) [30], American Zebra Optimization Algorithm (AZOA) [31], Cosine Swarm Algorithm (CSA) [32], Salp Swarm Algorithm (SSA) [33], Sine Cosine Algorithm (SCA) [34], Monarch Butterfly Optimization (MBO) [35], Mountain Gazelle Optimizer (MGO) [36], Golden Jackal Optimization (GJO) [37], Slime Mould Algorithm (SMA) [38], Multiverse Optimizer (MVO) [39], Archimedes Optimization Algorithm (AOA) [40], Gravitational Search Algorithm (GSA) [41], Equilibrium Optimizer (EO) [42], Kepler Optimization Algorithm (KOA) [43], Gradient Based Optimizer (GBO) [44], Teaching Learning Based Optimization (TLBO) [45], Poor and Rich Optimization (PRO) algorithm [46], Social Engineering Optimizer (SEO) [47], Student Psychology Based Optimization (SPBO) [48], Skill Optimization Algorithm (SOA) [49], and Group Learning Algorithm (GLA) [50]. Moreover, a number of well-known and recent publications have emerged to enhance the efficiency of existing MHAs by either implementing the innovative search technique or hybridizing specific applications into the original framework. Particularly, Eslami et al. [51] suggested a chaotic PSO (CPSO) in 2011 to promote diversification and avoid local optimal trapping in multi-machine power systems (MMPS) by simultaneously modifying the SVC damping regulator and PSS. In the same year [52], the improved PSO (IPSO) was introduced, which combines PSO with chaotic maps to find the ideal position of a power system stabilizer in a MMPS. In 2012 [53], an improved GSA was developed by the simultaneous coordination adjustment of the TCSC damping regulator and PSS in the multi-machine power system. In 2019, Truong et al. [54] proposed a multi-strategy SOS (QOCSOS) to accelerate convergence and improve the solution quality of SOS by integrating logistic map sequence and quasi-oppositional points. In 2022, Eslami et al. [55] proposed a productive hybrid optimization approach based on adaptive rat swarm optimization (ARSO) and pattern search (PS), which aids in enhancing the searching ability of PS by consistently extracting the parameters of photovoltaic models. In the same year, Li et al. [56] developed an improved binary PSO (IBPSO), a joint planning system of DGs and energy storage devices that uses bi-level programming for an active distribution network to mitigate the planning errors resulting from uncertainties in DGs outputs. Also, Turgut et al. [57] in 2022 introduced an improved arithmetic optimization algorithm (COAOA) to optimize the cost of a shell and tube condenser of the thermo-economic design when operating with different pairings of refrigerant mixtures. Liu et al. [58] introduced a chaotic simulated annealing MVO (CSAMVO) in 2022 by combining chaotic and simulated annealing mechanisms to balance the exploration and exploitation abilities of the classical MVO. In the same year, Hu et al. [59] suggested enhanced SMA (DFSMA) using a dispersed foraging approach to maintain the diversity of the population. In 2023, Zhang et al. [60] proposed a chaotic SSA with DE (CDESSA) to enhance the performance of SSA by chaotic initialization and DE approach. In the same year, Balu et al. [61] suggested an enhanced SPBO (QOCSPBO) by incorporating opposite points and a logistic chaotic map to address deciphering complex and global optimization problems. Furthermore, Gharehchopogh et al. [62] suggested an enhanced FFA (CQFFA) by employing twelve distinct chaotic maps and quasi-oppositional points to solve engineering applications.

Though these MHAs have shown excellent performance in confronting complex optimization problems, there are still certain limitations in balancing the exploration and exploitation abilities of the algorithms. For instance, the conventional GWO has the benefit of simple execution with minimal parameters, but it suffers from inadequate exploitation ability and stagnates in local optima when solving complex optimization problems. The PSO has the advantage of involving few parameter tunings but also has the disadvantage of local optimal entrapment in higher dimensions and a relatively sluggish convergence. In contrast, GA requires significant processing time and is difficult to optimize. The GJO technique has the advantages of being easy to implement, very reliable, and requiring less parameter adjustment; however, it also has the disadvantage of having imbalanced exploration and exploitation abilities, which can result in excessive exploitation and trapping in local optima. Also, GSA has the limitation of demanding an extensive set of control factors to reach global optima and has poor convergence precision. Furthermore, the recent TLBO offers the benefits of simple execution, lack of algorithm-specific parameters, and quick convergence, but it has the drawback of becoming trapped in local optimal regions. Similarly, SCA has the limitation of early convergence and poor accuracy, while WOA has the disadvantage of extensive computational time in addressing more intricate problems and sluggish performance to escape local optima. In this sense, researchers focused largely on further enhancing existing algorithms by incorporating multiple learning mechanisms (Refer [63], [64], [65], [66], [67], [68], [69], [70], [71]) or by hybridizing the major phases of distinct MHAs (Refer [72], [73], [74], [75], [76]). As a result, research is still ongoing to develop new MHAs or improve existing ones to address a variety of problems because of the limitations of the No Free Lunch (NFL) [77] theorem. The NFL theorem states that a particular optimization problem cannot tackle all the problems due to their inherent complexity and unique characteristics. This provides the research motivation for this study, which aims to further improve one of the most recent MHAs, the tunicate swarm algorithm. The Tunicate Swarm Algorithm (TSA) is a novel bio-inspired algorithm introduced by Kaur et al. in 2020 [23] that mimics the navigation and foraging patterns of deep-sea tunicates. The TSA has offered superior convergence accuracy and mobility performance. Furthermore, the algorithm has effectively been applied to address various constrained and unconstrained problems. However, the TSA has the drawback of being stuck in the local optima and having low diversification when dealing with highly complex problems. To overcome these drawbacks, numerous enhancements have been made to TSA by many researchers over a wide range of applications in order to strengthen the convergence rate and to maintain a proper balance between exploration and exploitation. For instance, Dogra et al. [78] employed TSA to enhance several performance indicators and optimize a process for prolonging network lifetime. Houssein et al. [79] in 2021 incorporated a local escaping operator with the standard TSA to enhance the solution accuracy of TSA. Li et al. [80] in the same year proposed an improved TSA (ITSA) to optimize three test systems and address dynamic economic and emission dispatch problems. Arabali et al. [81] introduced an adaptive TSA (ATSA) in 2022 to optimize the design of a shallow-spread foundation. In the same year, Gharehchopogh et al. [6] developed an improved TSA (QLGCTSA) by employing three mutation operators (Gaussian mutation, levy fight, and Cauchy mutation) to improve the global search ability of TSA. In 2023, Fathy et al. [82] proposed a modified TSA (MTSA) to increase the conversion efficiency of the PV array operating in partially shaded areas.

### 1.3 Contribution and paper organization

Although the aforementioned TSA variants improve the rate of convergence by preventing local optimal traps in highly challenging and multimodal functions, they persist in sluggish convergence and are prone to becoming trapped in local optimal regions when tackling some challenging problems. This serves as motivation for this study to propose an enhanced version of the TSA that addresses its inadequacies by introducing a novel technique into the conventional TSA. According to the NFL theorem [77], a novel and enhanced variant of TSA known as Quasi-Oppositional Chaotic TSA (QOCTSA) has been proposed in this study, which integrates Quasi-Oppositional Based Learning (QOBL) and chaotic local search (CLS) mechanisms into the framework of the conventional TSA. The CLS strategy is applied by employing ten distinct chaotic maps. The idea of QOBL assists in exploring the search area more intensively and enhances diversification, whereas chaotic functions emphasize the most promising regions of the search area and improve intensification. Thus, the proposed QOCTSA technique maintains a better balance between exploitation and exploration than the original TSA. The key advantages of the suggested QOCTSA are as follows: (a) rapid convergence; (b) higher probability of locating global optima; (c) requires less computing time; (d) achieves a superior balance between exploitation and exploration; and (e) is effective at addressing complex engineering design problems with constrained and unknown search bounds. These factors inspired the authors of the current study to propose a new enhanced TSA (QOCTSA) for solving global optimization problems.

The primary contribution of this present study is briefly described as follows:

• A novel and enhanced variant of TSA named Quasi-Oppositional Chaotic TSA (QOCTSA) is proposed for solving global optimization and engineering design problems.
• A suggested QOCTSA is a simultaneous integration of two search techniques (including QOBL and CLS strategies) into the conventional TSA.
• The QOBL technique helps in avoiding falling into local optima, thereby enhancing both diversification and convergence precision.
• The CLS strategy employs ten distinct chaotic maps to improve local search ability around the most promising regions and assist in increasing convergence rate, thereby efficiently exploiting the search area.
• The performance of QOCTSA is tested on a collection of thirty-three functions, comprising the twenty-three CEC2005 and ten CEC2019 test functions, to ensure its superiority. The statistical measures, the *t*-test and Wilcoxon rank-sum test, have been conducted to show the statistical significance of the presented QOCTSA approach.
• The experimental outcomes are tested against several other recently proposed and well-known MHAs to determine their efficacy. To demonstrate the problem-solving efficiency, the QOCTSA has also been applied to four real-world engineering design problems.
• The experimental and statistical findings exhibit the superiority and stability of the QOCTSA approach over other competing algorithms.

The subsequent sections of the paper are organized in the following manner: The brief outline of the TSA is illustrated in Section 2. Section 3 presents the developed QOCTSA approach with the QOBL and various chaotic map techniques. Section 4 provides a detailed discussion of the experimental results and analysis of the proposed work. The applicability of the QOCTSA technique to solving real-world applications is discussed in Section 5. Finally, Section 6 explains the conclusion and scope of future works in detail.

## 2 The standard tunicate swarm algorithm (TSA)

**Figure 2:**
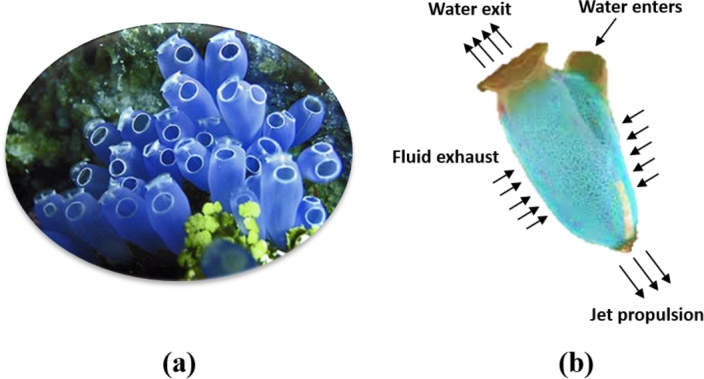
Jet propulsion behaviour of tunicates.

**Figure 3:**
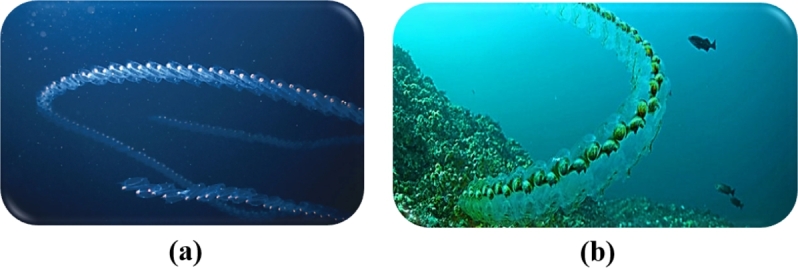
Swarming behaviour of tunicates.

In 2020, Kaur et al. [23] introduced a novel meta-heuristic paradigm called the Tunicate Swarm Algorithm (TSA) that mimics the social foraging behaviour of bioluminescent tunicates. Each tunicate is cylindrical in shape and exhibits a gelatinous tunic that assists in connecting all the other tunicates. However, TSA was inspired by two distinct behavioural patterns of tunicates in the deep sea, namely jet propulsion and swarm intelligence to locate the food source (i.e., the optimal solution). The visual representation of these patterns are depicted in Figure 2, Figure 3, respectively. To formulate the computational expression of the jet propulsion technique, it is necessary for a tunicate to satisfy the following constraints:

• Preventing collisions between the search individuals.
• Moving towards the direction of the best search individual.
• Converge to the region surrounding the best search individual.

In contrast, the swarm intelligence mechanism assists in updating the position of the tunicates based on the best optimal solutions. The mathematical formulations of these mechanisms are outlined in the subsequent subsections:

### 2.1 Preventing collisions between the search individuals

To prevent collision between the search individuals, the vector $\overset{\to }{A}$ is employed to determine the updated position of the search individual, as illustrated below:

(1) $$\overset{\to }{A}=\frac{\overset{\to }{G}}{\overset{\to }{M}}$$

(2) $$\overset{\to }{G}=r_{2}+r_{3}-\overset{\to }{F}$$

(3) $$\overset{\to }{F}=2⁎r_{1}$$

(4) $$\overset{\to }{M}=\lfloor P_{min}+r_{1}.(P_{max}-P_{min})\rfloor$$

where, the vectors $\overset{\to }{G}$ and $\overset{\to }{F}$ represent the gravitational force and the rate of water flow in the deep sea, respectively. The variables $r_{1}$, $r_{2}$, and $r_{3}$ are uniformly distributed random numbers within the range [0, 1]. Also, $\overset{\to }{M}$ indicates the social forces between the search individuals. Here, $P_{min}$ and $P_{max}$ are set to 1 and 4, respectively, and indicate the primary and secondary speeds of the search individuals.

### 2.2 Moving towards the direction of the best search individual

After preventing the collision between the search individuals, each should proceed towards the direction of the best search individual. The mathematical formulation of the optimization process for approaching the optimal search individual is defined as

(5) $$\overset{\to }{SD}=|F_{best}-rand⁎X(t)|$$

where, $\overset{\to }{SD}$ denotes the spatial distance from the tunicate to the food origin, $F_{best}$ depicts the position of food, X(t) represents the position of the tunicates, and rand∈[0,1].

### 2.3 Converge to the region surrounding the best search individual

The tunicates converge towards the position of the best search individual, as described below:

(6) $$X(t)=F_{best}+\overset{\to }{A}.\overset{\to }{SD},\;\;if\;rand\geq 0.5$$

(7) $$X(t)=F_{best}-\overset{\to }{A}.\overset{\to }{SD},\;\;if\;rand<0.5$$

where, X(t) indicates the updated position of each tunicate relative to the food position $F_{best}$.

### 2.4 Swarming behaviour of tunicates

In the swarm intelligence mechanism, the positions of tunicates are updated based on the positions of the first two best tunicates. This behaviour is illustrated as follows:

(8) $$X_{i}(t+1)=\left\{ \begin{array}{cc} \frac{X_{i}(t)+X_{i-1}(t+1)}{2+r_{1}},\; & if\;i>1\\ X_{i}(t),\; & if\;i=1 \end{array}\right.$$

where, i=1,2,...N, *N* is the population size of tunicates, $X_{i}(t+1)$ is the updated position of the current search individual of the next iteration, $X_{i-1}(t+1)$ is the position of the preceding search individual in the next iteration, and $X_{i}(t)$ is determined by Eqs. (6) and (7).

## 3 The proposed QOCTSA method

The conceptualization of the suggested QOCTSA is derived from the simultaneous incorporation of the QOBL and CLS by ten distinct chaotic map strategies into the standard TSA. And these strategies are elaborated in the following subsections.

### 3.1 Quasi-oppositional based learning (QOBL)

#### 3.1.1 A concept of QOBL

The classical meta-heuristic algorithms initiate the search process by generating a collection of initial populations in a random manner and iterate towards the optimal value. Therefore, the convergence rate relies on the proximity of the initial population to the optimal value. If the population generated at random deviates considerably from the optimal value, then the optimization process requires more time to converge, resulting in a very low convergence rate. In order to prevent this issue, Tizhoosh introduced the OBL [83] technique in 2005, which states that opposite numbers possess a greater chance of yielding an optimal solution compared to randomly selected numbers. The incorporation of meta-heuristics with OBL accelerates convergence and enhances solution precision. Furthermore, OBL has already been developed into QOBL [84], demonstrating that quasi-opposite numbers produce better optimal solutions than opposite numbers. The properties of QOBL are outlined below:

##### Opposite and quasi-opposite number

**Figure 4:**
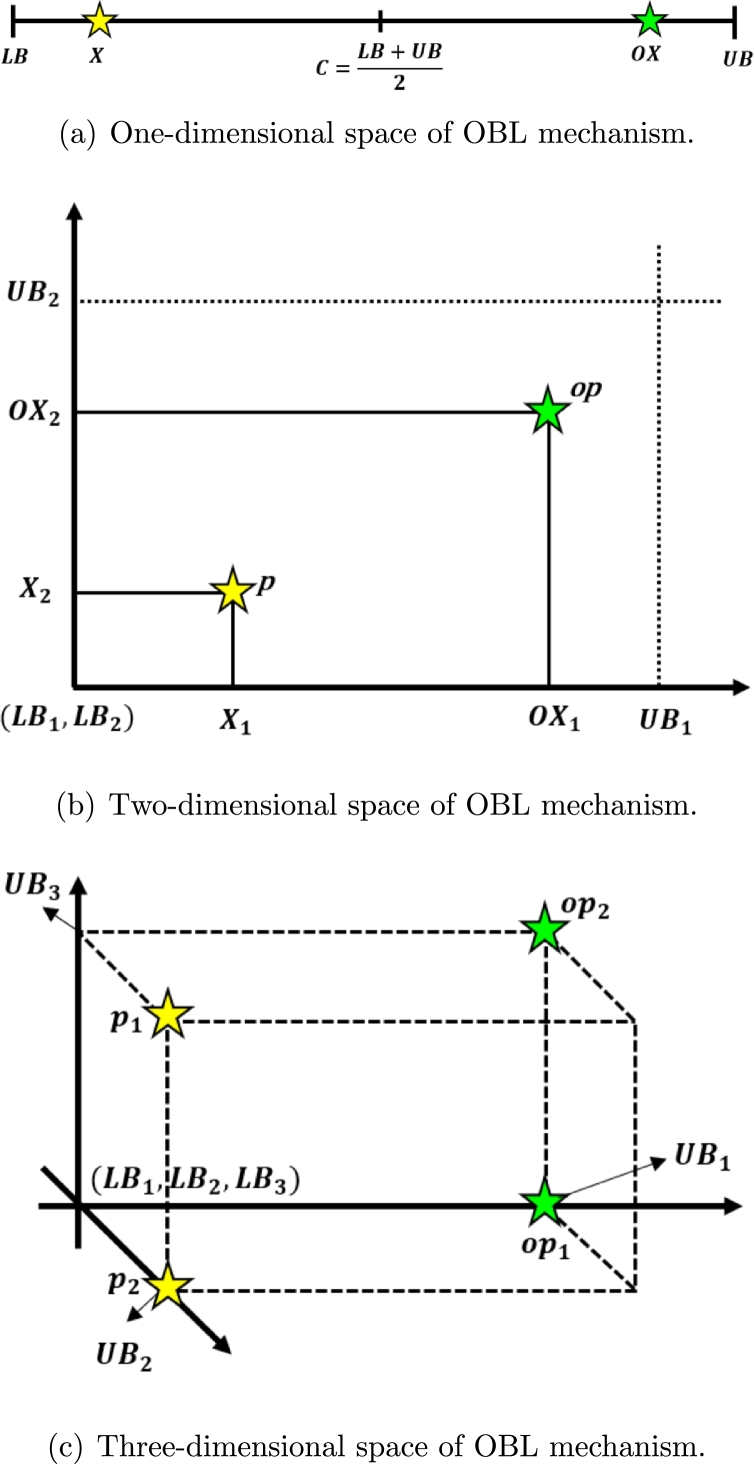
OBL mechanism in one, two, and three-dimensional spaces.

**Figure 5:**
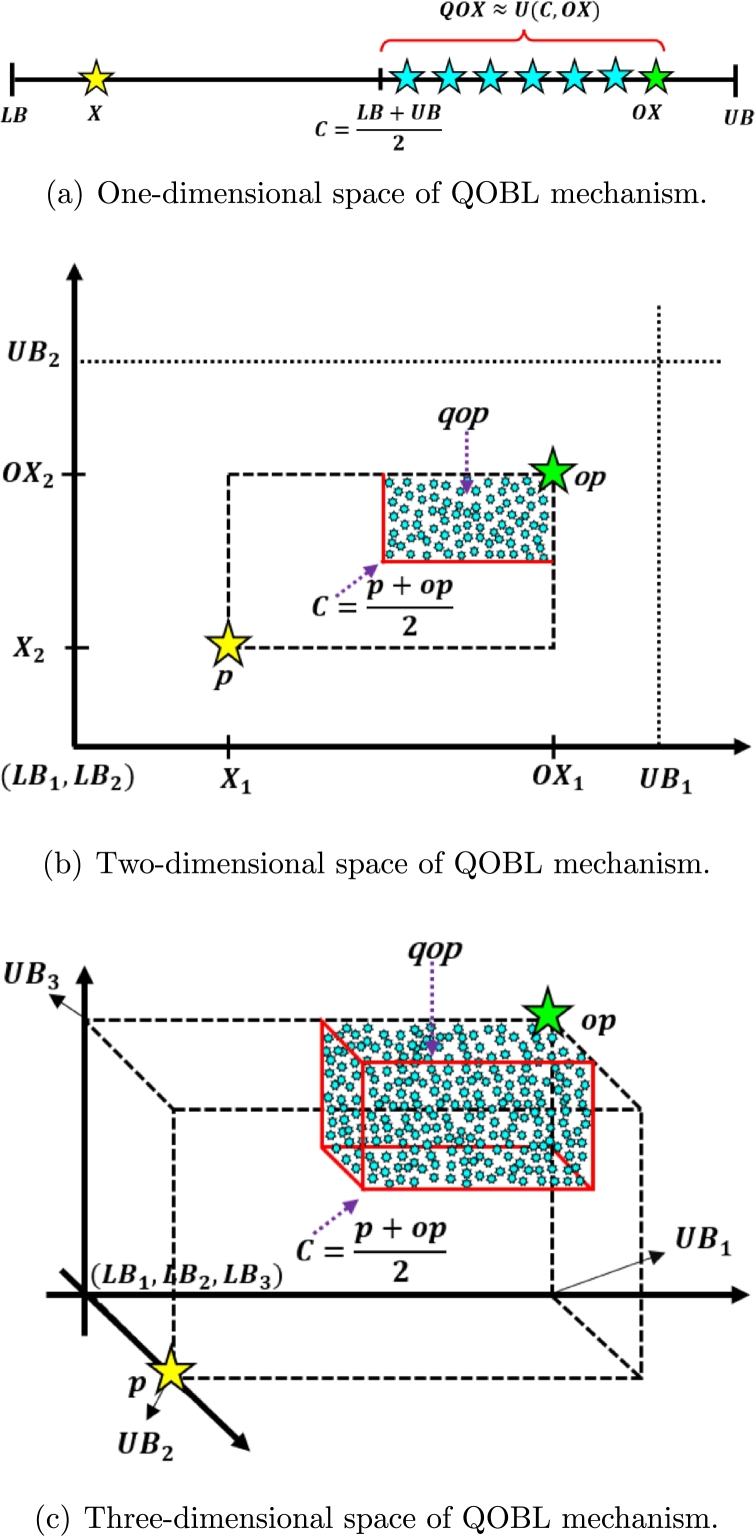
QOBL mechanism in one, two, and three-dimensional spaces.

For any random number X∈[LB,UB] in one-dimensional search space, the opposite number *OX* and the quasi-opposite number *QOX* of *X* are computed by Eqs. (9) and (10), respectively:

(9) OX=LB+UB−X

(10) QOX=rand(C,OX)

where, $C=\frac{LB+UB}{2}$, *LB* and *UB* indicate the lower and upper limits of the search space, respectively, and X∈R represent the initial population set. Figs. 4(a) to 4(c) and Figs. 5(a) to 5(c) depict the one, two, and three-dimensional representation of the OBL and QOBL mechanisms, respectively.

##### Opposite and quasi-opposite point

For any random point $X_{i}$$\left(x_{1},x_{2},...x_{n}\right)$ in *n*-dimensional search space, the opposite point $OX_{i}$$\left(ox_{1},ox_{2},...ox_{n}\right)$ and the quasi-opposite point $QOX_{i}$ $\left(qox_{1},qox_{2},...qox_{n}\right)$ of $X_{i}$ are computed by Eqs. (11) and (12), respectively:

(11) $$OX_{i}=LB_{i}+UB_{i}-X_{i}$$

(12) $$QOX_{i}=rand(C_{i},OX_{i})$$

where, $C_{i}=\frac{LB_{i}+UB_{i}}{2}$, X∈R and $X\in [LB_{i},UB_{i}]$, ∀i∈1,2,...n.

Since the quasi-opposite point exhibits a higher possibility of being close to the optimal value than the opposite point, QOBL is more effective at promoting exploration and accelerating convergence [62], [84]. In recent years, there has been extensive use of the QOBL technique in various MHAs in order to enhance their efficacy, such as quasi-oppositional DE [84], quasi-oppositional SOS [85], quasi-oppositional group search optimization [86], quasi-oppositional SSA [87], and quasi-oppositional TLBO [88].

#### 3.1.2 QOBL implementation in QOCTSA

In the QOCTSA, population initialization and generation jumping are performed by the QOBL technique. The population initialization by the QOBL approach generates both randomly produced and quasi-oppositional individuals. This technique aims to identify suitable solutions for the initial population by exploring the most prominent regions of the search area, thereby enhancing the effectiveness of the search procedure. Later, the process of generation jumping by the QOBL approach facilitates the algorithm to jump to a new solution that exhibits a higher level of fitness. The jumping parameter $\left(J_{r}\right)$, also known as jumping rate, helps in determining whether to maintain the present solution or switch to a quasi-opposing solution. Algorithm 1, Algorithm 2 present the pseudo-code for the QOBL technique in initializing tunicate population and generation jumping of the tunicates.

**Algorithm 1:**
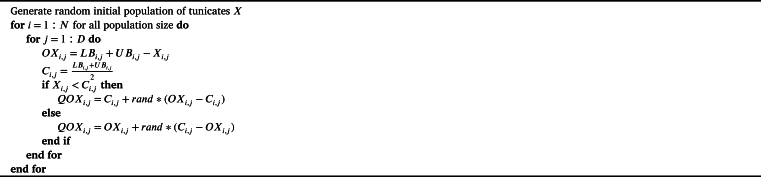
Pseudo-code of the QOBL technique in tunicates initialization.

**Algorithm 2:**
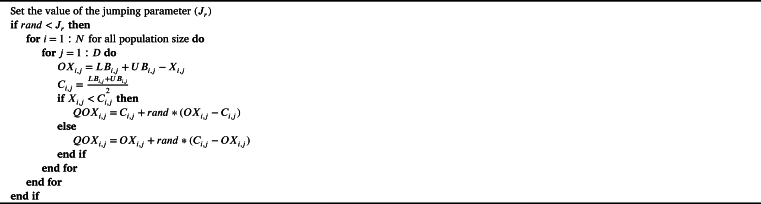
Pseudo-code of the QOBL technique in generation jumping.

### 3.2 Chaotic map functions

**Figure 6:**
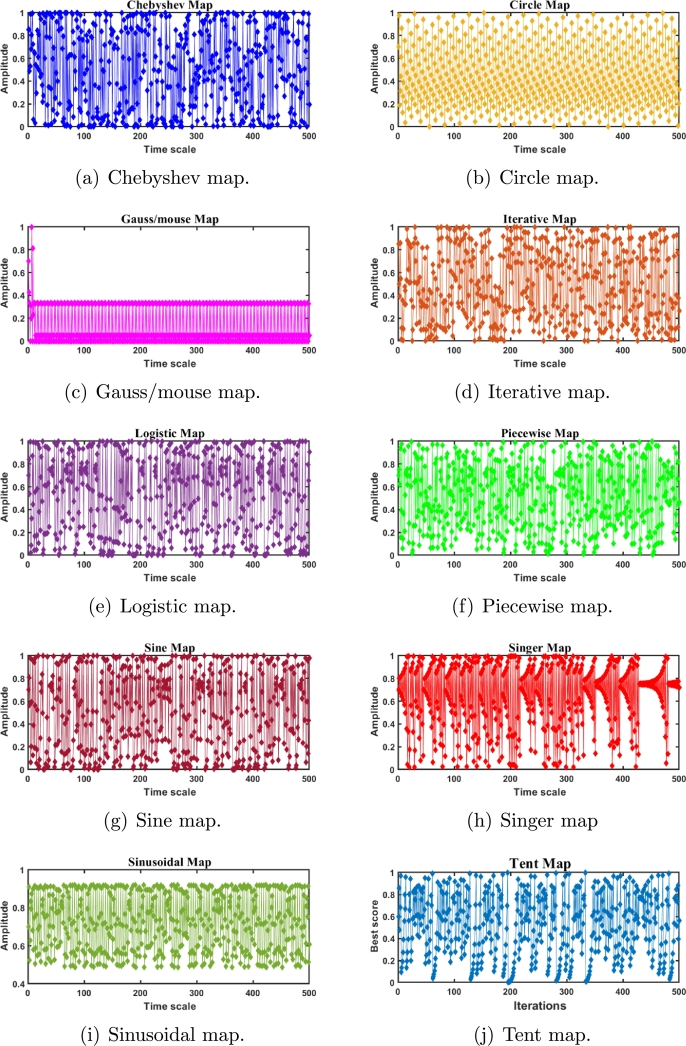
Stochastic behaviour of chaotic maps.

The notion chaos refers to non-linear, dynamic, and deterministic complex systems [89]. The unique characteristics such as ergodicity and non-repetition make the chaotic search method faster than the stochastic search method, which is probabilistic in nature [90]. Here, chaotic maps are employed to switch out existing random variables with chaotic ones. The values produced by the random function are not in a particular order, whereas the chaotic maps have the ability to produce random values in a specific order. Furthermore, there is no predetermined methodology for regulating the generation of subsequent random values. However, in the context of chaotic sequences, the random values are produced in an increasing manner, with relatively small differences between consecutive random values [91]. It is worth highlighting that the chaotic maps exhibit deterministic behaviour. Thus, the integration of chaotic maps into meta-heuristics can be considered an effective way to boost their performance by enhancing the exploitation of the most promising regions [62]. Some of the prominent chaos-enhanced MHAs are chaotic GWO [92], chaotic PSO [93], chaotic antlion algorithm [94], chaotic SSA [95], enhanced chaotic GWO [96], and chaotic KHA [97]. This work employs ten distinct chaotic maps, including the Chebyshev map, Circle map, Gauss/mouse map, Iterative map, Logistic map, Piecewise map, Sine map, Singer map, Sinusoidal map, and Tent map. The stochastic behaviour and the mathematical descriptions of these chaotic maps are outlined in Figs. 6(a) to 6(j) and Table 1, respectively.

**Table 1:** The formulations and the descriptions of the chaos maps.

**Map No.**	**Map Name**	**Mathematical Expression**	**Range**	**Name**
Map 1	Chebyshev Map	*x*_*i*+1_ = *cos*(*icos*^−1^(*x*_*i*_))	(-1,1)	QOCTSA1
Map 2	Circle Map	$x_{i+1}=mod(x_{i}+b-(\frac{a}{2\pi })sin(2\pi x_{i},1);a=0.5,b=0.2$	(0,1)	QOCTSA2
Map 3	Gauss/mouse Map	$x_{i+1}=\left\{ \begin{array}{cc} 1,\; & x_{i}=0\\ \frac{1}{mod(x_{i},1)},\; & otherwise \end{array}\right.$	(0,1)	QOCTSA3
Map 4	Iterative Map	$x_{i+1}=sin(\frac{a\pi }{x_{i}}),a=0.7$	(-1,1)	QOCTSA4
Map 5	Logistic Map	*x*_*i*+1_ = *ax*_*i*_(1 − *x*_*i*_),*a* = 4	(0,1)	QOCTSA5
Map 6	Piecewise Map	$x_{i+1}=\left\{ \begin{array}{cc} \frac{x_{i}}{P},\; & 0\leq x_{i}<P\\ \frac{x_{i}-P}{0.5-P},\; & P\leq x_{i}\leq \\ \frac{1-P-x_{i}}{0.5-P},\; & 0.5\leq x_{i}<1-P\\ 1-x_{i},\; & 1-P\leq x_{i}<1 \end{array}\right.,P=0.4$	(0,1)	QOCTSA6
Map 7	Sine Map	$x_{i+1}=\frac{a}{4}sin(\pi x_{i}),a=4$	(0,1)	QOCTSA7
Map 8	Singer Map	$x_{i+1}=\mu (7.86x_{i}-23.31x_{i}^{2}+28.75x_{i}^{3}-13.301875x_{i}^{4}),\mu =0.7$	(0,1)	QOCTSA8
Map 9	Sinusoidal Map	*x*_*i*+1_ = *ax*_*i*_*sin*(*πx*_*i*_),*a* = 4	(0,1)	QOCTSA9
Map 10	Tent Map	$x_{i+1}=\left\{ \begin{array}{cc} \frac{x_{i}}{0.7},\; & x_{i}<0.7\\ \frac{10}{3}(1-x_{i}),\; & x_{i}\geq 0.7 \end{array}\right.$	(0,1)	QOCTSA10

#### 3.2.1 Chaotic local search (CLS) strategy in QOCTSA

In this subsection, the chaotic maps are implemented as a CLS strategy to enhance the effectiveness of the conventional TSA. Also, this strategy inhibits the algorithm from getting trapped in the local optimal values and accelerates the convergence accuracy of the algorithm, enhancing exploitation. In the basic TSA, the potential for superior exploitation and exploration is achieved through the modifications of $\overset{\to }{A}$, $\overset{\to }{G}$, and $\overset{\to }{F}$, which are influenced by three primary parameters: $r_{1}$, $r_{2}$, and $r_{3}$ [23]. Furthermore, it is evident from Eq. (8) that $r_{1}$ is the primary parameter regulating the tunicate's updating position. Thus, the algorithm's convergence is dependent on Eq. (8), and it has an enormous effect on balancing exploitation and exploration. In this paper, the parameter $r_{1}$ of the conventional TSA is replaced by the chaotic variable, as shown in Eq. (13). The CLS strategy in the standard TSA can be defined as

(13) $$r_{1}=c(t)$$

(14) $$\begin{array}{c} \overset{\to }{A}=\frac{\overset{\to }{G}}{\overset{\to }{M}}\\ \overset{\to }{G}=r_{2}+r_{3}-\overset{\to }{F}\\ \overset{\to }{F}=2⁎c(t)\\ \overset{\to }{M}=\lfloor P_{min}+c(t).(P_{max}-P_{min})\rfloor \end{array}\}$$

(15) $$X_{i}(t+1)=\left\{ \begin{array}{cc} \frac{X_{i}(t)+X_{i-1}(t+1)}{2+c(t)},\; & if\;i>1\\ X_{i}(t),\; & if\;i=1 \end{array}\right.$$

Here, Eqs. (14) and (15) illustrate the process of updating the $r_{1}$ parameter in Eqs. (1) to (4) and the tunicate's updating position in Eq. (8), respectively, based on the utilization of a chaotic map. And the value c(t) represents the calculated value of chaos at the $t^{th}$ iteration.

### 3.3 The QOCTSA method

This section discusses the specifications of the proposed QOCTSA, comprising two novel techniques integrated into the TSA to enhance the efficiency and reliability of the proposed method:

• This study aims to implement the core concept of the QOBL technique into the recently developed TSA to further enhance the exploitation and exploration abilities of the algorithm.
• This QOBL strategy begins the search process by simultaneously generating the randomly produced and quasi-opposite solutions of tunicates in the initialization phase to select a collection of the best tunicates by exploring the most promising regions of the search domain.
• Later, the QOBL has been applied in generation jumping with the jumping parameter $\left(J_{r}\right)$, which forces the tunicates to jump to a new position in a chosen search area.
• This quasi-opposite population jumping has a greater possibility of producing the best tunicate than the existing one and assist in accelerating the reliability of the QOCTSA.
• Furthermore, another significant contribution of QOCTSA is to embed the CLS strategy of ten distinct well-known chaotic maps. This CLS technique prevents the QOCTSA from getting stuck in local optimal values and enhances its exploitation capability by extending its search into nearby regions.
• As a result, incorporating the QOBL technique with the CLS strategy into TSA significantly improves the convergence accuracy of the proposed QOCTSA by avoiding local optimal values with adequate random exploration.

**Figure 7:**
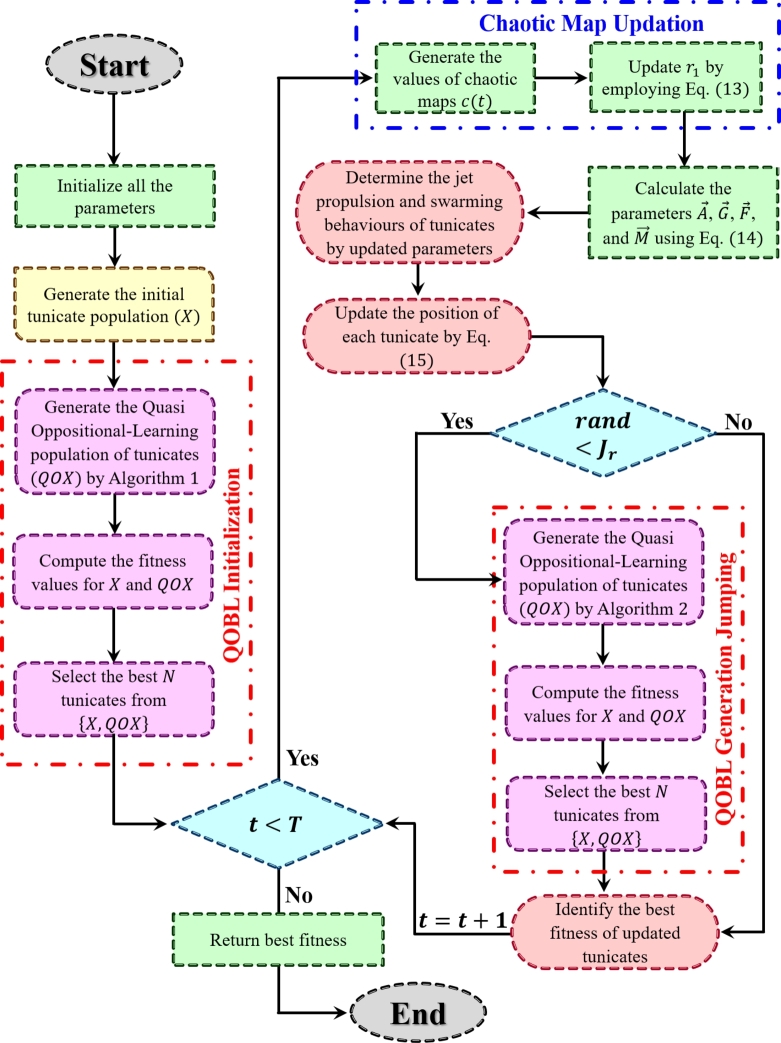
Flowchart of the proposed QOCTSA approach.

The detailed presentation of the search process of the developed QOCTSA is provided in Algorithm 3. The flowchart illustration of the proposed QOCTSA approach is also portrayed in Fig. 7.

**Algorithm 3:**
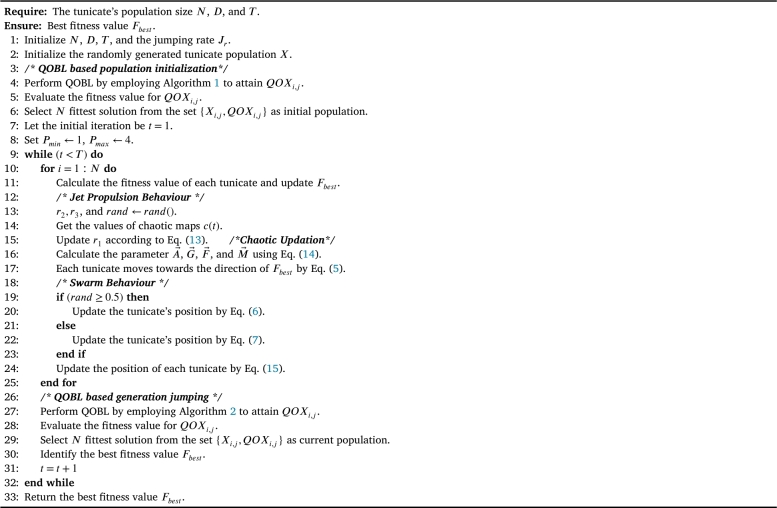
Pseudo-code of the proposed QOCTSA technique.

### 3.4 Computational complexity

An important component in evaluating an algorithm's performance is its computational complexity. In this study, the complexity is presented by employing the big-O notation [98]. Based on the pseudo-code mentioned above, the complexities of the standard TSA and proposed QOCTSA are discussed as follows:

#### 3.4.1 The standard TSA

• In TSA, initializing the population of each tunicate takes O(N×D) time, where *N* is the number of population size and *D* is the problem's dimensionality.
• The fitness evaluation of each tunicate requires O(N) time.
• The selection of each tunicate in TSA requires O(M) time, where *M* indicates the number of jet propulsion and swarming behaviour of tunicates for maintaining a better balance between exploration and exploitation.
• The position update of each tunicate in the standard TSA requires O(N×D) time.

In summary, the total computational time of the standard TSA is O(T×N×D×M), where *T* is the maximum number of iterations.

#### 3.4.2 The proposed QOCTSA

• In the proposed QOCTSA, initializing the population of tunicates takes O(N×D) time, where *N* is the number of tunicates and *D* is the problem's dimensionality.
• The calculation of each tunicate's quasi-opposite position in the initialization phase requires O(N×D) time. The fitness evaluation of each tunicate requires O(N) time.
• The chaotic local search (CLS) strategy requires O(N) time.
• The selection of each tunicate with CLS strategy in TSA requires O(N×M) time, where *M* indicates the number of jet propulsion, swarming behaviour and CLS strategy of the tunicates.
• The position update of each tunicate in the proposed QOCTSA requires O(N×D) time.
• In addition, the generation jumping of QOCTSA takes O(N) time to execute because it affects the entire tunicate population.

Hence, the overall computation time of QOCTSA for the entire algorithm is O(T×N×D×M)+O(N×T), where *T* is the maximum number of iterations. However, if the minimal complexities are omitted, the ultimate time complexity is O(T×N×D×M). Thus, the complexity remains consistent with the standard TSA and does not increase the computational time complexity.

Also, in the context of spatial complexity, the generation of *N* tunicates with the *D* dimension requires the largest amount of space in the initialization phase. Hence, the overall space complexity of TSA and QOCTSA is O(N×D). As a result, it can be concluded that the two algorithms are the same in terms of time and space complexity.

## 4 Experimental outcomes and discussion

This section tests the efficiency of the suggested QOCTSA method with two primary experiments. First, the efficacy of TSA and QOCTSAs employing ten distinct chaotic maps, such as the Chebyshev map, Circle map, Gauss/mouse map, Iterative map, Logistic map, Piecewise map, Sine map, Singer map, Sinusoidal map, and Tent map, is discussed, and the superiority of the chosen QOCTSA is established by the Wilcoxon rank-sum test. Then, the performance of the QOCTSA method is tested against the standard TSA [23], well-known algorithms like L-SHADE [19] and PSO [14], and the recently proposed algorithms, including WOA [28], SCA [34], SOA [27], and STOA [99]. The experimentations are performed on the operating system of Windows 11 with 8.00 GB RAM and a CPU of Intel(R) Core (TM) i5-1035G1 (1.00 GHz) and MATLAB R2022a.

### 4.1 CEC2005 and CEC2019 test functions

**Figure 8:**
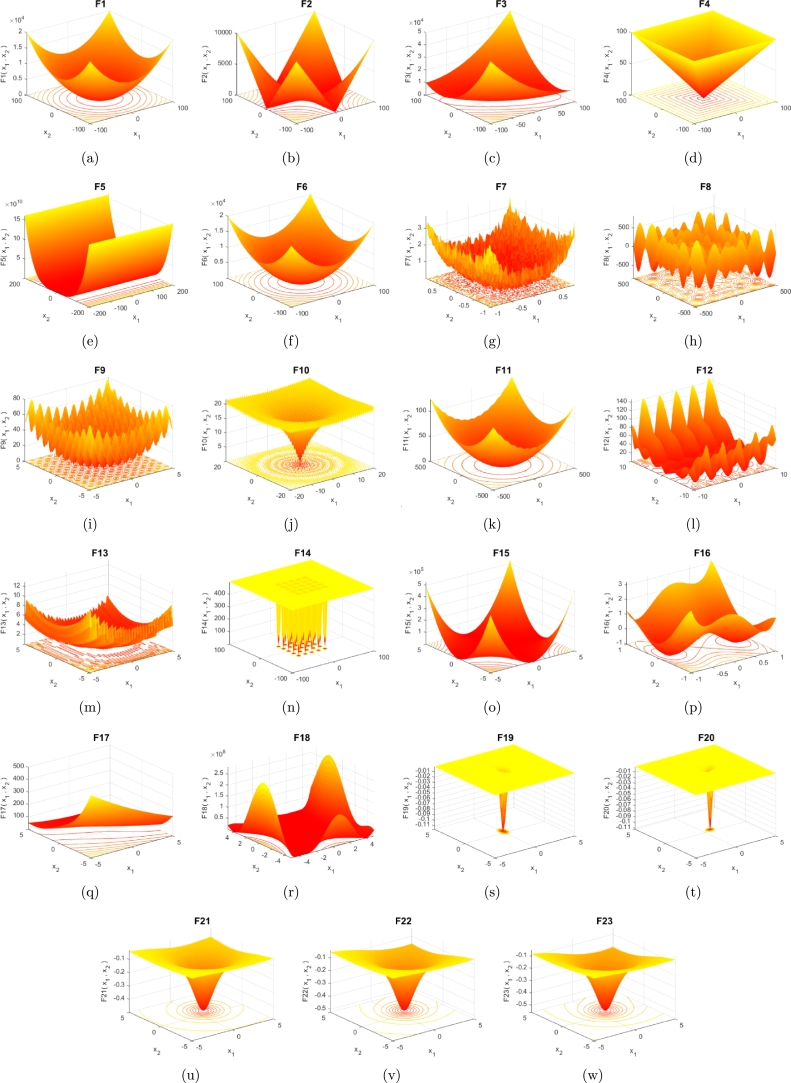
3D graphs of the CEC2005 benchmark functions.

In order to test the optimization capability of the presented QOCTSA, a diverse set of thirty-three test functions, comprising the twenty-three well-known CEC2005 [100] and ten CEC2019 [101] test functions, has been chosen. These test functions are categorized into unimodal (F1-F7), multimodal (F8-F13), fixed-dimensional multimodal (F14-F23), and CEC2019 (F24-F33) functions. Also, the test functions are designated by the letter ‘F,’ preceded by their corresponding numbers as F1, F2,…F33. The unimodal functions possess a unique global optimum and are intended to examine the exploitation ability of the algorithm. In contrast to the unimodal functions, the multimodal functions consist of multiple optimal values, with one being global optimum and the remaining are local optima. Therefore, the multimodal functions assist in examining the exploration ability of the algorithm. Finally, the fixed-dimensional multimodal functions can keep a proper balance between the exploitation and exploration abilities of the algorithm in both local and global searches since they have smaller dimensions and fewer local optima. The 3D graphs of these twenty-three CEC2005 [100] test functions are depicted in Figs. 8(a) to 8(w). To further evaluate the efficacy and robustness of the proposed algorithm, a highly complex set of ten CEC2019 [101] test functions has been employed. The optimization outcomes of these test functions of the proposed QOCTSA method are tested against L-SHADE [19], PSO [14], WOA [28], SCA [34], SOA [27], STOA [99], and TSA [23]. The evaluation metrics, such as the mean and standard deviation, are employed to determine the total performance of the compared algorithms. In addition, statistical experiments are performed to evaluate the superiority of one algorithm over others by analyzing the outcomes obtained from each trial run. The statistical experiments are essential due to the inherent stochastic nature of the MHAs. Hence, statistical tests such as the *t*-test and non-parametric Wilcoxon rank-sum tests are conducted to determine the reliability of the algorithms.

### 4.2 Parameter settings

In this study, the efficiency of the suggested QOCTSA method and L-SHADE [19], PSO [14], WOA [28], SCA [34], SOA [27], STOA [99], and standard TSA [23] are evaluated to determine the optimization ability of the CEC2005 [100] and CEC2019 [101] test functions. It is worth mentioning that each function employs a total of 30 search agents to conduct the search process. For a fair comparison, each function is executed a total of 30 times, with 15,000 function evaluations and a maximum limit of 500 iterations on different dimensions. Furthermore, the parameter settings for the other compared algorithms are reported in Table 2.

**Table 2:** The parameter settings of the various meta-heuristic algorithms.

**Algorithms**	**Parameters**	**Values**
L-SHADE	Memory size	5
	Archieve rate	1.4
	*p*_*best*_*rate*	0.11
PSO	Cognitive coefficient (*c*_1_)	1.5
	Social coefficient (*c*_2_)	2.0
	Inertia weight (*w*)	1
	Inertia damping weight (*w*_*damp*_)	0.99
WOA	*a*	[2, 0]
	*a*_2_	[−2, −1]
	Spiral factor (*b*)	1
SCA	No. of elites (*a*)	2
SOA	Control parameter (*A*)	[2, 0]
	*f*_*c*_	2
STOA	Control variable (*C*_*f*_)	2
AOA	Sensitive parameter (*α*)	5
	*μ*	0.5
TSA	Parameter *P*_*min*_	1
	Parameter *P*_*max*_	4
QOCTSA	Parameter *P*_*min*_	1
	Parameter *P*_*max*_	4
	Jumping probability (*J*_*r*_)	0.1

**Figure 9:**
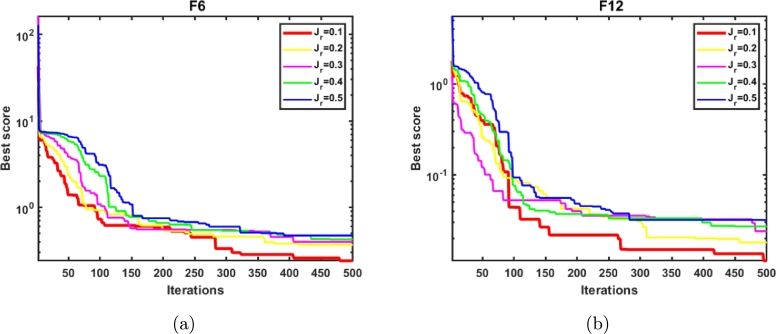
Analysis of the parameter jumping rate (*J*_*r*_).

The parameter $J_{r}$ helps in controlling the convergence rate of the suggested algorithm. A higher estimate of $J_{r}$ may rapidly reduce the population's diversity, which results in premature convergence. In order to determine an appropriate value of $J_{r}$, an experiment has been performed on the functions F6 and F12. The values of the jumping parameter $J_{r}$ varies from 0.1 to 0.5. The QOCTSA is evaluated by employing the functions F6 and F12 with 30 runs and 15,000 function evaluations. Fig. 9 outlines the statistical analysis of the varying $J_{r}$ values. From Figs. 9(a) and 9(b), it is noticed that the introduced method generates better outcomes when the value of $J_{r}$ is set to 0.1.

### 4.3 Performance of the QOCTSA with different chaotic maps

This section determines the most suitable chaotic map for each function by evaluating the performance of the QOCTSA and TSA algorithms using various chaotic maps. These evaluations are outlined by employing thirty-three different test functions in four distinct subsections, each of which is elaborated upon below.

#### 4.3.1 Unimodal test functions

**Figure 10:**
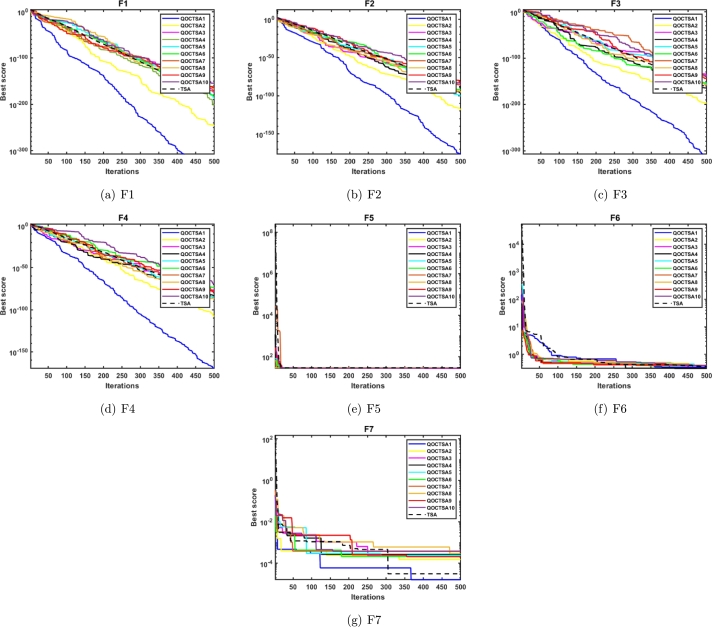
Convergence curves of the various chaotic maps on CEC2005 unimodal functions.

In this subsection, the efficiency of the suggested QOCTSA is evaluated on the standard CEC2005 unimodal (F1-F7) test functions. These functions possess a single optimal solution and are employed to evaluate the algorithm's exploitation ability. The detailed description of the unimodal test functions and the outcomes derived from the various chaotic maps are illustrated in Table 3, Table 4, respectively. In addition, Figs. 10(a) to 10(g) outline the convergence curves of the chaotic maps on the aforementioned test functions. Based on the statistical findings presented in Table 4, it is evident that the map QOCTSA3 exhibits a worse outcome than TSA, whereas the maps QOCTSA6, QOCTSA7, QOCTSA8, QOCTSA9, and QOCTSA10 yield mediocre results with respect to the mean and standard deviation. In contrast, the maps QOCTSA1, QOCTSA2, QOCTSA4, and QOCTSA5 achieve significantly superior outcomes for the majority of test functions compared to TSA. By analyzing the outcomes of Table 4 in more detail, it is observed that the Chebyshev map exhibits superior performance compared to the other maps. This is also confirmed by quantifying the number of successful outcomes for each chaotic map. The success rate is the total of the optimal outcomes for each map. Also in Table 4, the best chaotic map outcomes are bold-faced, while the best non-parametric Wilcoxon rank-sum test outcomes with *p*-values less than 0.05 relative to the standard TSA are underlined. The curves depicted in Fig. 10 demonstrate that the Chebyshev map exhibits a higher rate of competitive convergence than other chaotic maps. Hence, according to Table 4 and Fig. 10, it can be concluded that the integration of chaotic maps into the standard TSA algorithm has enhanced its performance in solving CEC2005 unimodal test functions.

**Table 3:** Details of the unimodal test functions.

**Name**	**Functions**	**Dimension**	**Range**	$f_{min}$
F1	$f(x)=\sum_{i=1}^{n}z_{i}^{2}$	30	[−100, 100]	0
F2	$f(x)=\sum_{i=1}^{n}|z_{i}|+\prod_{i=1}^{n}|z_{i}|$	30	[−10, 10]	0
F3	$f(x)=\sum_{i=1}^{n}{\left(\sum_{j=1}^{i}z_{j}\right)}^{2}$	30	[−100, 100]	0
F4	*f*(*x*)=*max*_*i*_{|*z*_*i*_|,1 ≤ *i* ≤ *n*}	30	[−100, 100]	0
F5	$f(x)=\sum_{i=1}^{n-1}[100{\left(z_{i+1}-z_{i}^{2}\right)}^{2}+{\left(z_{i}-1\right)}^{2}]$	30	[−30, 30]	0
F6	$f(x)=\sum_{i=1}^{n}{\left(|z_{i}+0.5|\right)}^{2}$	30	[−100, 100]	0
F7	$f(x)=\sum_{i=1}^{n}iz_{i}^{4}+random[0,1)$	30	[−1.28, 1.28]	0

**Table 4:** Statistical outcomes of various chaotic maps on CEC2005 unimodal test functions.

**Func.**		**TSA**	**QOCTSA1**	**QOCTSA2**	**QOCTSA3**	**QOCTSA4**	**QOCTSA5**	**QOCTSA6**	**QOCTSA7**	**QOCTSA8**	**QOCTSA9**	**QOCTSA10**
**F1**	**Mean**	5.301E-180	**0**	2.578E-219	5.187E-167	5.742E-194	1.014E-183	1.196E-176	1.242E-161	5.104E-163	3.877E-167	1.264E-156
	**Std**	**0**	0	0	0	0	0	0	0	0	0	0
	*p***-values**		1.777E-06	1.779E-06	3.111E-01	1.776E-06	1.392E-05	8.365E-05	3.546E-02	7.384E-03	1.501E-02	2.309E-01
**F2**	**Mean**	4.791E-85	**2.654E-163**	9.593E-116	2.394E-77	1.174E-92	8.177E-100	7.739E-90	4.387E-87	3.844E-97	1.037E-86	9.213E-85
	**Std**	2.624E-84	**0**	4.758E-115	1.311E-76	6.404E-92	3.317E-99	4.236E-89	2.401E-86	2.000E-96	5.682E-86	5.046E-84
	*p***-values**		1.778E-06	1.776E-06	3.962E-01	1.392E-05	1.779E-06	1.778E-04	2.656E-05	1.779E-06	2.909E-05	1.080E-04
**F3**	**Mean**	9.718E-160	**6.425E-305**	1.086E-190	1.893E-133	1.472E-161	9.848E-156	4.223E-152	1.242E-131	6.173E-148	1.533E-144	1.596E-131
	**Std**	0	**0**	0	7.037E-133	0	0	2.313E-151	6.801E-131	3.381E-147	8.395E-144	8.740E-131
	*p***-values**		1.779E-06	1.778E-06	7.850E-03	2.909E-05	3.183E-05	1.418E-02	5.204E-01	3.729E-02	4.774E-02	7.227E-01
**F4**	**Mean**	3.090E-84	**8.642E-154**	2.465E-106	4.326E-77	5.390E-84	2.659E-84	8.295E-76	2.097E-71	6.736E-83	3.758E-74	8.567E-62
	**Std**	1.427E-83	**4.714E-153**	1.332E-105	1.740E-76	2.419E-83	1.456E-83	4.542E-75	1.148E-70	3.657E-82	2.057E-73	4.692E-61
	*p***-values**		1.779E-06	1.779E-06	3.848E-01	3.383E-04	3.183E-05	7.227E-01	6.179E-01	3.383E-04	1.031E-01	7.074E-01
**F5**	**Mean**	2.804E+01	2.724E+01	2.749E+01	**2.717E+01**	2.744E+01	2.747E+01	2.740E+01	2.732E+01	2.752E+01	2.746E+01	2.769E+01
	**Std**	8.960E-01	**6.260E-01**	9.861E-01	6.955E-01	8.115E-01	8.282E-01	7.615E-01	6.735E-01	8.522E-01	7.730E-01	9.190E-01
	*p***-values**		3.007E-03	1.418E-02	4.622E-04	6.525E-03	3.921E-02	7.384E-03	3.435E-03	2.212E-02	2.739E-02	1.121E-01
**F6**	**Mean**	3.507E-01	3.434E-01	3.821E-01	3.984E-01	**3.352E-01**	3.861E-01	3.851E-01	3.908E-01	3.950E-01	3.886E-01	3.920E-01
	**Std**	7.660E-02	7.676E-02	7.078E-02	5.451E-02	5.950E-02	5.665E-02	5.253E-02	5.745E-02	5.671E-02	6.255E-02	**4.812E-02**
	*p***-values**		6.325E-01	5.511E-02	1.588E-02	5.204E-01	6.054E-02	3.729E-02	3.546E-02	1.878E-02	6.640E-02	9.411E-03
**F7**	**Mean**	1.025E-04	**5.650E-05**	1.375E-04	2.595E-04	2.603E-04	2.210E-04	2.039E-04	3.367E-04	2.829E-04	2.137E-04	2.955E-04
	**Std**	1.088E-04	**5.388E-05**	1.086E-04	2.545E-04	3.219E-04	2.548E-04	2.091E-04	5.503E-04	2.578E-04	2.290E-04	2.932E-04
	*p***-values**		2.598E-02	2.825E-01	9.990E-03	9.990E-03	2.463E-02	4.121E-02	3.918E-03	2.270E-04	6.640E-02	1.263E-02

**Sum of Best Outcomes**		0	**5**	0	1	1	0	0	0	0	0	0

#### 4.3.2 Multimodal test functions

**Figure 11:**
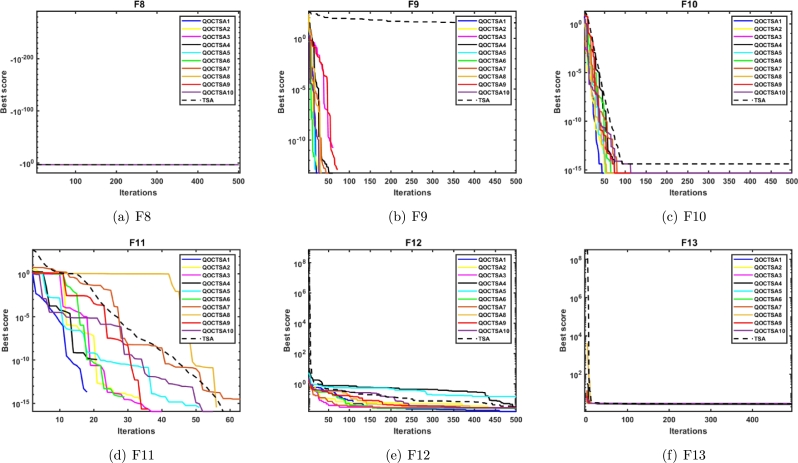
Convergence curves of the various chaotic maps on CEC2005 multimodal functions.

In this subsection, the performance of the introduced QOCTSA is tested on the classical CEC2005 multimodal (F8-F13) test functions. These functions comprise multiple optimal values that help in determining the algorithm's exploration ability. The detailed depiction of the multimodal test functions and the statistical outcomes from the various chaotic maps are reported in Table 5, Table 6, respectively. Also, Figs. 11(a) to 11(f) depict the convergence curves of the chaotic maps of these test functions. By evaluating the statistical outcomes of Table 6, it is obvious that the outcomes of all the chaotic variants of the TSA algorithm for functions F9 to F11 are identical. Moreover, it is observed that all the maps have attained optimal values for functions F9 and F11. Furthermore, the chaotic maps on F12 achieved promising outcomes for all the test functions, excluding the QOCTSA5 and QOCTSA6 maps. The map QOCTSA4 outperforms all other maps on the majority of test functions. Also, Table 6 reveals that the Chebyshev map and Iterative map have outperformed all other maps. Fig. 11 illustrates that all chaotic TSA maps have attained more optimal values and superior convergence compared to the conventional TSA algorithm. It also confirms that the Chebyshev map has a high rate of convergence. In addition, Table 6 bolds the best chaotic map outcomes and underlines the best Wilcoxon rank-sum test outcomes with *p*-values less than 5% significance level, in contrast to the classical TSA. From Table 6 and Fig. 11, it can be deduced that the incorporation of the chaotic maps into the basic TSA algorithm has improved its performance when dealing with CEC2005 multimodal test functions.

**Table 5:** Details of the multimodal test functions.

**Name**	**Functions**	**Dimension**	**Range**	$f_{min}$
F8	$f(x)=-\sum_{i=1}^{n}z_{i}sin(\sqrt{\left|z_{i}\right|})$	30	[−500, 500]	−12569.5
F9	$f(x)=\sum_{i=1}^{n}[z_{i}^{n}-10cos(2\pi z_{i})+10]$	30	[−5.12, 5.12]	0
F10	$f(x)=-20exp(-0.2\sqrt{\frac{1}{n}}\sum_{i=1}^{n}z_{i}^{2})-exp(\frac{1}{n}\sum_{i=1}^{n}cos(2\pi z_{i}))+20+e$	30	[−32, 32]	0
F11	$f(x)=\frac{1}{4000}\sum_{i=1}^{n}z_{i}^{2}-\prod_{i=1}^{n}cos(\frac{z_{i}}{\sqrt{i}})+1$	30	[−600, 600]	0
F12	$f(x)=\frac{\pi }{n}\{10sin(\pi y_{1})+\sum_{i=1}^{n}{\left(y_{i}-1\right)}^{2}[1+10sin^{2}(\pi y_{i+1})]+{\left(y_{n}-1\right)}^{2}\}+\sum_{i=1}^{n}u(z_{i},10,100,4)$			
	$y_{i}=1+\frac{z_{i}+1}{4}$			
	$u(z_{i},a,k,m)=\left\{ \begin{array}{cc} k{\left(z_{i}-a\right)}^{m}\; & z_{i}>a\\ 0\; & -a<z_{i}<a\\ k{\left(-z_{i}-a\right)}^{m}\; & z_{i}<-a \end{array}\right.$	30	[−50, 50]	0
F13	$f(x)=0.1\{sin^{2}(3\pi z_{1})+\sum_{i=1}^{n}{\left(z_{i}-1\right)}^{2}[1+sin^{2}(3\pi z_{i}+1)]+{\left(z_{n}-1\right)}^{2}[1+sin^{2}(2\pi z_{n})]\}+\sum_{i=1}^{n}u(z_{i},5,100,4)$	30	[−50, 50]	0

**Table 6:** Statistical outcomes of various chaotic maps on CEC2005 multimodal test functions.

**Func.**		**TSA**	**QOCTSA1**	**QOCTSA2**	**QOCTSA3**	**QOCTSA4**	**QOCTSA5**	**QOCTSA6**	**QOCTSA7**	**QOCTSA8**	**QOCTSA9**	**QOCTSA10**
**F8**	**Mean**	−**4.130E+03**	−4.002E+03	−3.897E+03	−3.747E+03	−3.825E+03	−3.692E+03	−3.848E+03	−3.779E+03	−3.765E+03	−3.884E+03	−3.868E+03
	**Std**	3.551E+02	6.437E+02	4.101E+02	2.552E+02	4.240E+02	**2.401E+02**	4.202E+02	4.523E+02	2.954E+02	3.470E+02	4.603E+02
	*p***-values**		1.373E-01	4.330E-02	1.930E-04	1.060E-02	1.392E-05	5.405E-03	3.007E-03	9.805E-04	1.776E-02	2.212E-02
**F9**	**Mean**	5.552E+01	**0**	**0**	**0**	**0**	**0**	**0**	**0**	**0**	**0**	**0**
	**Std**	5.809E+01	**0**	**0**	**0**	**0**	**0**	**0**	**0**	**0**	**0**	**0**
	*p***-values**		9.207E-05	9.207E-05	9.207E-05	9.207E-05	9.207E-05	9.207E-05	9.207E-05	9.207E-05	9.207E-05	9.207E-05
**F10**	**Mean**	3.997E-15	**4.441E-16**	**4.441E-16**	**4.441E-16**	**4.441E-16**	**4.441E-16**	**4.441E-16**	**4.441E-16**	**4.441E-16**	**4.441E-16**	**4.441E-16**
	**Std**	2.407E-30	**3.009E-31**	**3.009E-31**	**3.009E-31**	**3.009E-31**	**3.009E-31**	**3.009E-31**	**3.009E-31**	**3.009E-31**	**3.009E-31**	**3.009E-31**
	*p***-values**		4.467E-08	4.467E-08	4.467E-08	4.467E-08	4.467E-08	4.467E-08	4.467E-08	4.467E-08	4.467E-08	4.467E-08
**F11**	**Mean**	**0**	**0**	**0**	**0**	**0**	**0**	**0**	**0**	**0**	**0**	**0**
	**Std**	**0**	**0**	**0**	**0**	**0**	**0**	**0**	**0**	**0**	**0**	**0**
	*p***-values**		NA	NA	NA	NA	NA	NA	NA	NA	NA	NA
**F12**	**Mean**	3.813E-02	**2.015E-02**	3.040E-02	2.999E-02	3.645E-02	4.984E-02	4.552E-02	2.358E-02	2.507E-02	3.266E-02	2.329E-02
	**Std**	5.445E-02	**4.667E-03**	6.301E-02	3.607E-02	7.615E-02	1.296E-01	8.151E-02	2.773E-02	3.165E-02	6.538E-02	2.321E-02
	*p***-values**		1.621E-03	7.850E-03	3.546E-02	1.339E-02	2.212E-02	2.390E-01	1.134E-03	2.456E-03	2.463E-02	1.865E-03
**F13**	**Mean**	2.798E+00	2.850E+00	2.925E+00	2.860E+00	**2.785E+00**	2.912E+00	2.867E+00	2.811E+00	2.864E+00	2.918E+00	2.859E+00
	**Std**	2.227E-01	3.494E-01	**1.880E-01**	2.770E-01	3.372E-01	2.186E-01	2.313E-01	3.157E-01	2.720E-01	1.932E-01	2.590E-01
	*p***-values**		3.111E-01	1.075E-01	6.922E-01	6.621E-01	2.558E-01	5.893E-01	9.467E-01	6.179E-01	2.918E-01	8.977E-01

**Sum of Best Outcomes**		2	**4**	3	3	**4**	3	3	3	3	3	3

#### 4.3.3 Fixed-dimensional multimodal test functions

**Figure 12:**
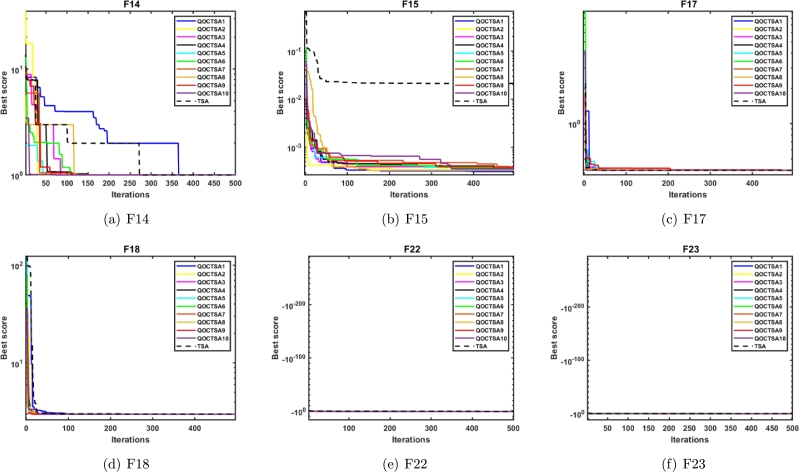
Convergence curves of the various chaotic maps on CEC2005 fixed-dimensional multimodal functions.

This subsection explains the outcomes of the introduced QOCTSA on fixed-dimensional multimodal (F14-F23) functions. The dimensionality of these test functions remains constant and cannot be altered. Table 7 illustrates the description of the fixed-dimensional multimodal functions. The experimental analysis and the convergence graphs of the distinct chaotic maps utilized in the TSA are presented in Table 8 and Fig. 12, respectively. The experimental outcomes in Table 8 demonstrate that the chaotic maps used in the conventional TSA yield superior and promising outcomes for the specified functions except F14 in comparison to the basic TSA. Also, it is obvious from Table 8 that the Chebyshev map outperforms all other maps in terms of mean and standard deviation. Furthermore, in Table 8, the most favourable outcomes obtained from the various chaotic maps are indicated by bold formatting. Similarly, the superior outcomes derived from the non-parametric Wilcoxon rank-sum test are highlighted by underlining. The curves depicted in Figs. 12(a) to 12(f) illustrate that the outcomes of the different chaotic maps exhibit a high degree of similarity with minor differences. Hence, it can be concluded from Table 8 and Fig. 12 that the incorporation of chaotic maps into the basic TSA algorithm has enhanced its effectiveness when dealing with CEC2005 fixed-dimensional multimodal test functions.

**Table 7:** Details of the fixed-dimensional multimodal test functions.

**Name**	**Functions**	**Dimension**	**Range**	$f_{min}$
F14	$f(x)={\left[\frac{1}{500}+\sum_{j=1}^{25}\frac{1}{j+\sum_{i=1}^{2}{\left(z_{i}-a_{ij}\right)}^{6}}\right]}^{-1}$	2	[−65.53,65.53]	1
F15	$f(x)=\sum_{i=1}^{n}|a_{i}-\frac{z_{1}(b_{i}^{2}+b_{i}z_{2})}{b_{i}^{2}+b_{i}z_{3}+z_{4}}{|}^{2}$	4	[−5,5]	0.0003
F16	$f(x)=4z_{1}^{2}-2.1z_{1}^{4}+\frac{1}{3}z_{1}^{6}+z_{1}z_{2}-4z_{2}^{2}+4z_{2}^{4}$	2	[−5,5]	−1.0316
F17	$f(x)={\left(z_{2}-\frac{5.1}{4\pi^{2}}z_{1}^{2}+\frac{5}{\pi }z_{1}-1\right)}^{2}+10(1-\frac{1}{8\pi })cosz_{1}+10$	2	[−5,5]	0.398
F18	$f(x)=[1+{\left(z_{1}+z_{2}+1\right)}^{2}(19-14z_{1}+3z_{1}^{2}-14z_{2}+6z_{1}z_{2}+3z_{2}^{2})]\times [30{\left(2z_{1}-3z_{2}\right)}^{2}(18-32z_{1}+12z_{1}^{2}+48z_{2}-36z_{1}z_{2}+27z_{2}^{2})]$	2	[−2,2]	3.000
F19	$f(x)=-\sum_{i=1}^{4}c_{i}exp(-\sum_{j=1}^{3}a_{ij}{\left(z_{j}-p_{ij}\right)}^{2})$	3	[0,1]	−3.86
F20	$f(x)=\sum_{i=1}^{4}c_{i}exp(-\sum_{j=1}^{6}a_{ij}{\left(z_{j}-p_{ij}\right)}^{2})$	6	[0,1]	−3.32
F21	$f(x)=\sum_{i=1}^{5}|(z_{i}-a_{i}){\left(z_{i}-a_{i}\right)}^{T}+c_{i}{|}^{-1}$	4	[0,10]	−10.1532
F22	$f(x)=\sum_{i=1}^{7}|(z_{i}-a_{i}){\left(z_{i}-a_{i}\right)}^{T}+c_{i}{|}^{-1}$	4	[0,10]	−10.4028
F23	$f(x)=\sum_{i=1}^{1}0|(z_{i}-a_{i}){\left(z_{i}-a_{i}\right)}^{T}+c_{i}{|}^{-1}$	4	[0,10]	−10.5363

**Table 8:** Statistical outcomes of various chaotic maps on CEC2005 fixed-dimensional multimodal test functions.

**Func.**		**TSA**	**QOCTSA1**	**QOCTSA2**	**QOCTSA3**	**QOCTSA4**	**QOCTSA5**	**QOCTSA6**	**QOCTSA7**	**QOCTSA8**	**QOCTSA9**	**QOCTSA10**
**F14**	**Mean**	**4.249E+00**	7.615E+00	8.002E+00	7.612E+00	7.223E+00	6.445E+00	6.445E+00	7.612E+00	6.445E+00	7.612E+00	8.002E+00
	**Std**	**4.906E+00**	5.880E+00	5.816E+00	5.883E+00	5.923E+00	5.923E+00	5.923E+00	5.883E+00	5.923E+00	5.883E+00	5.816E+00
	*p***-values**		1.258E-02	1.785E-02	8.050E-02	1.404E-01	2.415E-01	3.418E-01	1.258E-02	3.568E-01	6.649E-02	2.339E-02
**F15**	**Mean**	7.210E-03	3.254E-04	1.044E-03	1.668E-03	1.684E-03	**3.222E-04**	3.511E-04	1.034E-03	3.967E-04	3.529E-04	1.039E-03
	**Std**	9.631E-03	1.832E-05	3.652E-03	5.086E-03	5.084E-03	**1.477E-05**	6.030E-05	3.652E-03	2.189E-04	5.856E-05	3.651E-03
	*p***-values**		3.262E-06	5.920E-05	5.893E-06	1.638E-04	3.604E-06	1.049E-05	3.383E-04	1.678E-05	1.154E-05	1.930E-04
**F16**	**Mean**	−1.032E+00	−**1.032E+00**	−1.032E+00	-1.032E+00	-1.032E+00	−1.032E+00	−1.032E+00	−1.032E+00	−1.032E+00	−1.032E+00	−1.032E+00
	**Std**	1.174E-05	1.544E-05	8.353E-06	1.182E-05	**4.752E-06**	7.457E-06	5.116E-06	1.194E-05	1.477E-05	9.435E-06	9.399E-06
	*p***-values**		6.770E-01	8.815E-01	8.977E-01	2.004E-01	6.621E-01	5.893E-01	8.491E-01	9.140E-01	8.170E-01	6.035E-01
**F17**	**Mean**	3.985E-01	**3.981E-01**	3.990E-01	3.985E-01	3.991E-01	3.987E-01	3.987E-01	3.984E-01	3.985E-01	3.986E-01	3.985E-01
	**Std**	7.014E-04	**2.599E-04**	1.352E-03	9.716E-04	1.859E-03	8.932E-04	1.033E-03	7.638E-04	1.004E-03	1.038E-03	8.539E-04
	*p***-values**		1.984E-02	2.335E-02	6.325E-01	1.796E-01	3.962E-01	4.940E-01	3.848E-01	9.467E-01	9.795E-01	9.795E-01
**F18**	**Mean**	3.002E+00	**3.000E+00**	3.000E+00	3.000E+00	3.000E+00	3.000E+00	3.000E+00	3.000E+00	3.000E+00	3.000E+00	3.000E+00
	**Std**	2.204E-03	**2.042E-04**	1.991E-04	3.762E-04	2.675E-04	2.611E-04	3.341E-04	3.974E-04	3.385E-04	2.201E-04	3.618E-04
	*p***-values**		2.019E-05	9.540E-06	4.847E-06	4.163E-05	2.656E-05	5.920E-05	5.425E-05	6.459E-05	2.019E-05	5.425E-05
**F19**	**Mean**	−3.859E+00	−**3.860E+00**	−3.857E+00	−3.857E+00	-3.858E+00	−3.860E+00	−3.858E+00	−3.858E+00	−3.859E+00	−3.859E+00	−3.858E+00
	**Std**	3.355E-03	**2.636E-03**	3.237E-03	3.017E-03	3.477E-03	3.267E-03	3.380E-03	3.405E-03	3.385E-03	3.643E-03	3.484E-03
	*p***-values**		3.201E-02	1.485E-01	1.267E-01	9.303E-01	1.124E-02	5.752E-01	3.520E-01	3.013E-01	6.922E-01	9.631E-01
**F20**	**Mean**	−3.178E+00	−**3.303E+00**	−3.235E+00	−3.255E+00	-3.205E+00	−3.214E+00	−3.223E+00	−3.209E+00	−3.204E+00	−3.231E+00	−3.192E+00
	**Std**	1.899E-01	**1.761E-02**	9.945E-02	6.236E-02	1.185E-01	8.292E-02	9.418E-02	9.852E-02	1.177E-01	8.851E-02	9.767E-02
	*p***-values**		3.660E-04	2.825E-01	9.469E-02	7.382E-01	8.815E-01	7.074E-01	5.893E-01	9.959E-01	4.314E-01	8.977E-01
**F21**	**Mean**	−7.055E+00	−**9.125E+00**	−7.522E+00	−7.598E+00	-7.833E+00	−7.430E+00	−7.818E+00	−7.695E+00	−7.734E+00	−7.704E+00	−8.012E+00
	**Std**	2.107E+00	**6.835E-01**	1.677E+00	1.544E+00	1.258E+00	1.827E+00	1.034E+00	1.537E+00	1.360E+00	1.737E+00	1.350E+00
	*p***-values**		9.111E-05	3.962E-01	1.730E-01	1.428E-01	6.035E-01	9.882E-02	1.604E-01	1.796E-01	1.933E-01	3.370E-02
**F22**	**Mean**	−6.375E+00	−**9.314E+00**	−7.715E+00	−7.675E+00	-7.432E+00	−6.886E+00	−7.741E+00	−7.411E+00	−7.700E+00	−7.872E+00	−8.381E+00
	**Std**	**2.411E+00**	5.378E-01	1.371E+00	1.457E+00	1.698E+00	2.193E+00	1.736E+00	1.932E+00	1.446E+00	1.843E+00	1.088E+00
	*p***-values**		4.847E-06	6.341E-02	2.335E-02	6.950E-02	5.752E-01	4.547E-02	1.319E-01	6.640E-02	1.124E-02	9.111E-04
**F23**	**Mean**	−5.651E+00	−**9.309E+00**	−8.167E+00	−7.655E+00	-7.606E+00	−7.981E+00	−7.183E+00	−7.488E+00	−7.825E+00	−7.886E+00	−7.928E+00
	**Std**	3.040E+00	**6.429E-01**	1.172E+00	1.805E+00	2.247E+00	1.695E+00	2.397E+00	2.226E+00	1.786E+00	1.461E+00	1.641E+00
	*p***-values**		2.952E-06	1.511E-03	8.862E-03	3.215E-03	2.812E-03	2.886E-02	2.212E-02	5.072E-03	4.461E-03	3.435E-03

**Sum of Best Outcomes**		1	**7**	1	0	0	1	0	0	0	0	0

#### 4.3.4 CEC2019 test functions

**Figure 13:**
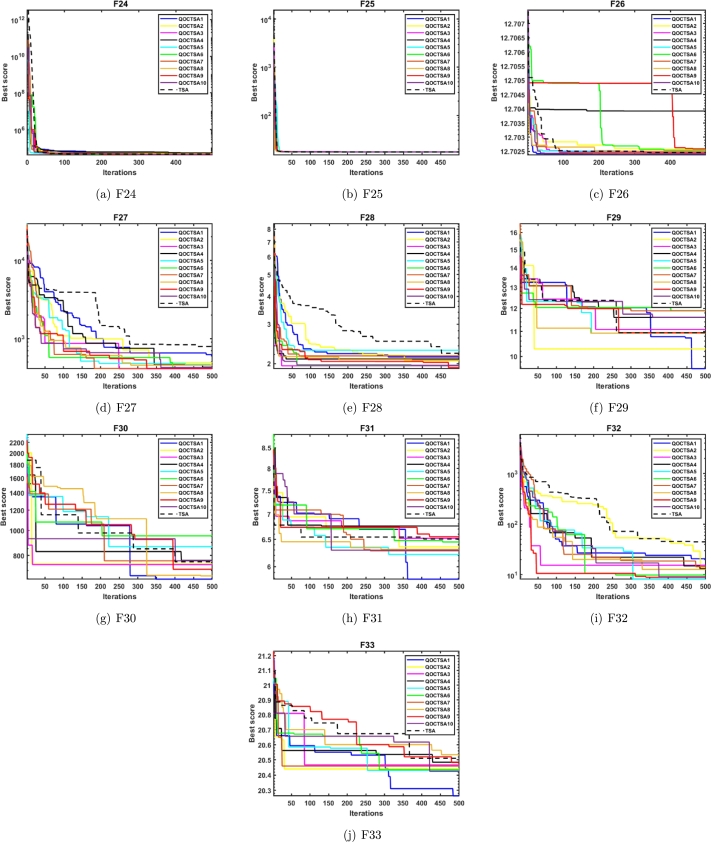
Convergence curves of the various chaotic maps on CEC2019 test functions.

This subsection examines the effectiveness of the proposed QOCTSA on a set of challenging CEC2019 (F24-F33) test functions. It comprises ten distinct functions, each characterized by its own unique dimensions and search range. The functions from F24 to F26 have different dimensions, whereas the ranges of all the remaining functions are consistently bounded within the interval [-100, 100]. The comprehensive description of the CEC2019 test functions is outlined in Table 9. The statistical findings and the convergence graphs of the ten chaotic maps with TSA are reported in Table 10 and Fig. 13, respectively. According to the experimental outcomes provided in Table 10, it is evident that the map QOCTSA1 outperforms the majority of functions. In terms of the mean and standard deviation, it has been illustrated that the map QOCTSA9 has exhibited superior outcomes for functions F24 and F32, while the remaining maps have demonstrated mediocre results, excluding QOCTSA5 and QOCTSA8. By examining the outcomes of Table 10 in more detail reveals that both the Chebyshev and Sinusoidal maps perform better than all other maps. Also, this is tested by estimating the total number of successful outcomes for each chaotic map. Furthermore, Table 10 highlights the best possible outcomes of various chaotic maps in bold text and underlines the statistically significant outcomes of the Wilcoxon rank-sum test with a significance level of less than 5% in comparison to the TSA. The graphs illustrated in Figs. 13(a) to 13(j) demonstrate that the outcomes of various chaotic maps converge more effectively than the standard TSA.

**Table 9:** CEC2019 test functions.

**Name**	**Functions**	**Dimension**	**Range**	$f_{min}$
F24	Storn's Chebyshev Polynomial Fitting Problem	9	[−8192, 8192]	1
F25	Inverse Hilbert Matrix Problem	16	[−16,384, 16,384]	1
F26	Lennard-Jones Minimum Energy Cluster	18	[−4, 4]	1
F27	Shifted Rotated Rastrigin's Function	10	[−100, 100]	1
F28	Shifted Rotated Grienwangk's Function	10	[−100, 100]	1
F29	Shifted Rotated Weierstrass Function	10	[−100, 100]	1
F30	Modified Schwefel's Function	10	[−100, 100]	1
F31	Expanded Schaffer's Function	10	[−100, 100]	1
F32	Shifted Rotated Happy Cat Function	10	[−100, 100]	1
F33	Shifted Rotated Ackley Function	10	[−100, 100]	1

**Table 10:** Statistical outcomes of various chaotic maps on CEC2019 test functions.

**Func.**		**TSA**	**QOCTSA1**	**QOCTSA2**	**QOCTSA3**	**QOCTSA4**	**QOCTSA5**	**QOCTSA6**	**QOCTSA7**	**QOCTSA8**	**QOCTSA9**	**QOCTSA10**
**F24**	**Mean**	5.296E+04	5.000E+04	5.137E+04	4.915E+04	4.781E+04	4.909E+04	4.958E+04	4.789E+04	4.787E+04	**4.693E+04**	4.878E+04
	**Std**	8.002E+03	5.554E+03	6.036E+03	6.168E+03	6.603E+03	6.452E+03	6.514E+03	5.837E+03	6.048E+03	**5.108E+03**	7.312E+03
	*p***-values**		1.863E-01	5.204E-01	9.469E-02	1.418E-02	6.640E-02	1.168E-01	4.330E-02	2.598E-02	2.812E-03	7.606E-02
**F25**	**Mean**	1.740E+01	**1.738E+01**	1.740E+01	1.743E+01	1.741E+01	1.742E+01	1.743E+01	1.744E+01	1.744E+01	1.745E+01	1.745E+01
	**Std**	5.558E-02	**1.668E-02**	3.600E-02	6.222E-02	3.082E-02	3.711E-02	6.183E-02	5.649E-02	4.853E-02	6.910E-02	7.393E-02
	*p***-values**		9.882E-02	1.680E-02	4.278E-04	2.629E-03	6.274E-04	3.808E-05	5.816E-04	9.111E-05	1.638E-04	3.383E-04
**F26**	**Mean**	**1.270E+01**	**1.270E+01**	**1.270E+01**	**1.270E+01**	**1.270E+01**	**1.270E+01**	**1.270E+01**	**1.270E+01**	**1.270E+01**	**1.270E+01**	**1.270E+01**
	**Std**	1.104E-05	**6.065E-06**	8.453E-06	1.818E-05	2.820E-04	4.553E-04	1.752E-05	2.086E-05	2.752E-04	1.048E-05	1.996E-05
	*p***-values**		8.977E-01	2.473E-01	3.040E-02	5.405E-03	7.857E-04	6.941E-03	1.192E-02	2.461E-04	4.461E-03	8.462E-04
**F27**	**Mean**	8.032E+02	6.099E+02	4.761E+02	4.377E+02	4.514E+02	4.459E+02	4.416E+02	4.374E+02	**4.183E+02**	4.441E+02	4.562E+02
	**Std**	5.973E+02	4.111E+02	1.478E+02	1.101E+02	1.192E+02	1.118E+02	1.429E+02	8.361E+01	**1.016E+02**	8.980E+01	1.099E+02
	*p***-values**		1.796E-01	5.255E-02	7.850E-03	1.263E-02	1.192E-02	6.525E-03	7.384E-03	7.857E-04	6.525E-03	4.758E-03
**F28**	**Mean**	2.382E+00	2.292E+00	2.021E+00	2.005E+00	2.001E+00	**1.977E+00**	1.977E+00	2.003E+00	1.992E+00	1.990E+00	1.978E+00
	**Std**	5.014E-01	4.078E-01	1.205E-01	**6.988E-02**	1.269E-01	8.742E-02	1.021E-01	1.199E-01	1.018E-01	8.153E-02	9.018E-02
	*p***-values**		2.230E-01	4.549E-05	4.393E-06	1.529E-05	1.678E-05	1.529E-05	1.841E-05	1.268E-05	8.671E-06	5.346E-06
**F29**	**Mean**	1.095E+01	**1.079E+01**	1.088E+01	1.092E+01	1.111E+01	1.082E+01	1.085E+01	1.097E+01	1.098E+01	1.083E+01	1.099E+01
	**Std**	6.059E-01	8.831E-01	6.792E-01	8.316E-01	5.884E-01	7.916E-01	7.270E-01	6.279E-01	6.492E-01	7.815E-01	**5.467E-01**
	*p***-values**		2.645E-01	8.011E-01	7.538E-01	3.520E-01	5.474E-01	8.170E-01	9.303E-01	7.227E-01	6.621E-01	9.631E-01
**F30**	**Mean**	7.350E+02	**6.056E+02**	7.455E+02	7.875E+02	7.394E+02	7.798E+02	8.023E+02	7.952E+02	7.366E+02	7.657E+02	7.558E+02
	**Std**	2.253E+02	1.800E+02	1.785E+02	1.748E+02	**1.701E+02**	1.957E+02	2.191E+02	2.193E+02	1.835E+02	2.096E+02	1.934E+02
	*p***-values**		4.121E-02	9.959E-01	2.558E-01	6.179E-01	2.230E-01	1.543E-01	3.520E-01	9.631E-01	7.382E-01	6.922E-01
**F31**	**Mean**	6.367E+00	**5.797E+00**	6.269E+00	6.156E+00	6.052E+00	5.997E+00	6.027E+00	6.080E+00	6.146E+00	6.109E+00	6.185E+00
	**Std**	**3.658E-01**	3.661E-01	4.632E-01	4.687E-01	5.199E-01	4.338E-01	4.254E-01	4.456E-01	4.756E-01	4.552E-01	4.572E-01
	*p***-values**		1.192E-02	6.922E-01	1.666E-01	9.411E-03	6.942E-03	5.757E-03	4.774E-02	1.121E-01	2.463E-02	1.031E-01
**F32**	**Mean**	4.231E+01	2.093E+01	1.036E+01	1.269E+01	1.301E+01	8.855E+00	9.554E+00	1.188E+01	1.156E+01	**8.281E+00**	9.425E+00
	**Std**	7.767E+01	4.468E+01	5.655E+00	9.306E+00	1.045E+01	5.302E+00	8.260E+00	7.821E+00	8.147E+00	**3.716E+00**	6.145E+00
	*p***-values**		3.415E-01	8.684E-02	1.031E-01	2.004E-01	7.384E-0	4.330E-0	1.485E-01	2.734E-01	1.984E-02	3.921E-0
**F33**	**Mean**	2.048E+01	**2.023E+01**	2.045E+01	2.046E+01	2.047E+01	2.044E+01	2.044E+01	2.046E+01	2.047E+01	2.046E+01	2.043E+01
	**Std**	**8.093E-02**	1.177E-01	8.862E-02	9.797E-02	1.042E-01	1.470E-01	1.286E-01	1.090E-01	9.147E-02	1.001E-01	1.349E-01
	*p***-values**		2.181E-06	1.543E-01	4.811E-01	7.695E-01	4.436E-01	1.543E-01	4.436E-01	6.621E-01	4.684E-01	1.267E-01

**Sum of Best Outcomes**		1	**6**	1	1	1	2	1	1	2	2	1

Based on these findings, it can be deduced that the integration of the chaotic sequence values into the fundamental parameters of the dynamic equations has significantly improved the efficiency of the TSA algorithm by accelerating global optima and preventing local optima. Also, the experimental analysis of the success rate of these CEC2005 [100] and CEC2019 [101] test functions stated that the Chebyshev map has outperformed other chaotic maps for the majority of test functions. Thus, the Chebyshev map is chosen as the most suitable map. The subsequent section will provide a more extensive analysis of the chosen map.

### 4.4 Performance of the QOCTSA with other meta-heuristic algorithms

The primary goal of this section is to test the effectiveness of the proposed QOCTSA with Chebyshev map to that of other state-of-the-art algorithms, including L-SHADE [19], PSO [14], WOA [28], SCA [34], SOA [27], STOA [99], and the standard TSA [23]. Table 2 discloses the parameter settings for these algorithms. The statistical measurements like the average and standard deviation of the thirty-three distinct test functions are reported in Table 11. From Table 11, it is evident that the proposed QOCTSA approach has achieved superior average fitness outcomes compared to the other algorithms for the majority of test functions. Among the seven unimodal test functions (F1-F7), QOCTSA performs more efficiently on six functions, except F6. In particular, the test function F1 has attained a global optimum value when compared to other algorithms. This demonstrates that the proposed QOCTSA technique has significant exploitation ability. In the multimodal functions (F8-F13), QOCTSA outperforms on the functions F9 and F11, whereas the functions F10 and F11 have achieved competitive outcomes. By comparing the QOCTSA with the other competing algorithms, it can be concluded that the incorporation of quasi-opposite solutions in TSA can effectively assist in escaping the local optimal solutions. However, the functions (F14-F23) in fixed-dimensional multimodal problems reveal that the QOCTSA method yields more promising mean outcomes for the functions F15, F17, and F19-F23 than other competitive algorithms. Also, in the CEC2019 test functions (F24-F33), the proposed QOCTSA demonstrates superior performance in comparison to the other algorithms, specifically for functions F24, F29, F31, and F33.

**Table 11:** Outcomes of the CEC2005 and CEC2019 test functions (the best outcomes are emphasized in bold text).

**Functions**		**L-SHADE**	**PSO**	**WOA**	**SCA**	**SOA**	**STOA**	**TSA**	**QOCTSA**
**F1**	**Mean**	2.057E-10	3.295E-02	3.821E-74	1.023E+01	5.423E-12	6.319E+00	5.301E-180	**0**
	**Std**	4.927E-10	3.750E-02	9.996E-74	2.515E+01	9.697E-12	3.123E+00	0	0
	*t***-values**	−**2.287E+00**	−**4.813E+00**	−**2.094E+00**	−**2.228E+00**	−**3.063E+00**	−**3.594E+00**	NA	
**F2**	**Mean**	1.631E-05	4.625E-01	2.954E-51	2.221E-02	2.207E-08	9.169E-06	4.791E-85	**2.654E-163**
	**Std**	3.918E-05	3.094E-01	1.093E-50	4.979E-02	1.846E-08	8.148E-06	2.624E-84	0
	*t***-values**	−**2.280E+00**	−**8.187E+00**	−***1.480E+00***	−**2.444E+00**	−**6.550E+00**	−**6.164E+00**	−***1.000E+00***	
**F3**	**Mean**	2.368E+00	9.646E+01	4.679E+04	7.846E+03	5.786E-05	5.186E-02	9.718E-160	**6.425E-305**
	**Std**	2.635E+00	3.033E+01	1.200E+04	6.278E+03	1.191E-04	6.394E-02	0	0
	*t***-values**	−**4.922E+00**	−**1.742E+01**	−**2.137E+01**	−**6.845E+00**	−**2.661E+00**	−**4.443E+00**	NA	
**F4**	**Mean**	7.339E+00	1.218E+00	4.324E+01	3.509E+01	3.286E-03	6.731E-02	3.090E-84	**8.642E-154**
	**Std**	2.498E+00	2.149E-01	2.891E+01	1.152E+01	6.481E-03	1.089E-01	1.427E-83	4.714E-153
	*t***-values**	−**1.609E+01**	−**3.104E+01**	−**8.192E+00**	−**1.668E+01**	−**2.777E+00**	−**3.386E+00**	−***1.186E+00***	
**F5**	**Mean**	3.068E+01	1.253E+02	2.796E+01	2.037E+04	2.831E+01	2.838E+01	2.804E+01	2.724E+01
	**Std**	2.093E+01	7.864E+01	4.020E-01	2.626E+04	5.822E-01	3.699E-01	8.960E-01	6.260E-01
	*t***-values**	−***9.011E-01***	−**6.828E+00**	−**5.369E+00**	−**4.244E+00**	−**6.852E+00**	−**8.597E+00**	−**4.006E+00**	
**F6**	**Mean**	5.139E-02	2.989E-02	4.270E-01	3.136E+01	3.229E+00	2.638E+00	3.507E-01	3.434E-01
	**Std**	4.509E-02	2.220E-02	2.443E-01	6.554E+01	4.947E-01	5.184E-01	7.660E-02	7.676E-02
	*t***-values**	1.797E+01	2.149E+01	−**1.788E+00**	−**2.592E+00**	−**3.157E+01**	−**2.398E+01**	−**3.691E-01**	
**F7**	**Mean**	3.316E-02	2.345E-01	4.242E-03	1.377E-01	2.823E-03	6.962E-03	1.025E-04	**5.650E-05**
	**Std**	1.475E-02	1.015E-01	4.085E-03	1.055E-01	1.888E-03	5.280E-03	1.088E-04	5.388E-05
	*t***-values**	−**1.230E+01**	−**1.265E+01**	−**5.611E+00**	−**7.146E+00**	−**8.023E+00**	−**7.163E+00**	−***4.508E-01***	
**F8**	**Mean**	−1.240E+04	−6.007E+03	−1.036E+04	−3.777E+03	−5.277E+03	−5.142E+03	−4.130E+03	−4.002E+03
	**Std**	1.448E+02	1.428E+03	1.851E+03	2.613E+02	8.813E+02	4.450E+02	3.551E+02	6.437E+02
	*t***-values**	6.972E+01	7.009E+00	1.777E+01	−**1.774E+00**	6.397E+00	7.976E+00	***9.551E-01***	
**F9**	**Mean**	9.950E-02	7.451E+01	***0***	3.818E+01	2.634E+00	6.931E+00	5.552E+01	***0***
	**Std**	5.450E-01	1.778E+01	0	3.474E+01	5.207E+00	7.063E+00	5.809E+01	0
	*t***-values**	−***1.000E+00***	−**2.295E+01**	NA	−**6.018E+00**	−**2.770E+00**	−**5.375E+00**	−**5.234E+00**	
**F10**	**Mean**	2.495E+00	8.105E-01	4.707E-15	1.153E+01	1.996E+01	1.996E+01	3.997E-15	**4.441E-16**
	**Std**	7.137E-01	6.294E-01	3.415E-15	9.489E+00	1.324E-03	1.332E-03	2.407E-30	3.009E-31
	*t***-values**	−**1.915E+01**	−**7.053E+00**	−**6.837E+00**	−**6.657E+00**	−**8.260E+04**	−**8.211E+04**	−**8.022E+15**	
**F11**	**Mean**	2.006E-02	1.469E-02	***0***	1.103E+00	1.275E-02	2.756E-02	***0***	***0***
	**Std**	2.316E-02	1.144E-02	0	5.558E-01	2.451E-02	3.814E-02	0	0
	*t***-values**	−**4.745E+00**	−**7.031E+00**	NA	−**1.087E+01**	−**2.850E+00**	−**3.958E+00**	NA	
**F12**	**Mean**	3.748E-01	2.526E-02	2.899E-01	9.561E+04	3.454E-01	2.224E-01	3.813E-02	**2.015E-02**
	**Std**	7.458E-01	8.730E-02	1.450E+00	3.140E+05	1.798E-01	1.142E-01	5.445E-02	4.667E-03
	*t***-values**	−**2.604E+00**	−***3.199E-01***	−***1.019E+00***	−***1.668E+00***	−**9.906E+00**	−**9.692E+00**	−**1.802E+00**	
**F13**	**Mean**	1.033E-01	**1.673E-02**	5.233E-01	1.424E+05	2.022E+00	1.977E+00	2.798E+00	2.850E+00
	**Std**	3.288E-01	1.600E-02	2.789E-01	4.970E+05	2.210E-01	2.534E-01	2.227E-01	3.494E-01
	*t***-values**	3.136E+01	4.437E+01	2.851E+01	−***1.569E+00***	1.097E+01	1.108E+01	***6.885E-01***	
**F14**	**Mean**	**9.980E-01**	4.507E+00	2.700E+00	2.185E+00	2.476E+00	2.018E+00	4.249E+00	7.615E+00
	**Std**	9.857E-14	3.090E+00	3.037E+00	1.896E+00	2.459E+00	1.898E+00	4.906E+00	5.880E+00
	*t***-values**	6.163E+00	2.563E+00	4.067E+00	4.813E+00	4.416E+00	4.961E+00	2.407E+00	
**F15**	**Mean**	3.380E-04	8.268E-04	6.229E-04	1.068E-03	1.167E-03	1.758E-03	7.210E-03	**3.254E-04**
	**Std**	1.672E-04	2.068E-04	2.652E-04	3.736E-04	2.355E-04	3.525E-03	9.631E-03	1.832E-05
	*t***-values**	−***4.122E-01***	−**1.323E+01**	−**6.129E+00**	−**1.088E+01**	−**1.951E+01**	−**2.225E+00**	−**3.916E+00**	
**F16**	**Mean**	−***1.032E+00***	−***1.032E+00***	−***1.032E+00***	−***1.032E+00***	−***1.032E+00***	−***1.032E+00***	−***1.032E+00***	−***1.032E+00***
	**Std**	4.091E-08	0	5.536E-09	4.885E-05	9.272E-07	1.776E-06	1.174E-05	1.544E-05
	*t***-values**	4.765E+00	4.767E+00	4.768E+00	−**4.004E+00**	4.332E+00	4.036E+00	***7.988E-01***	
**F17**	**Mean**	3.979E-01	3.979E-01	3.979E-01	4.002E-01	3.982E-01	3.982E-01	3.985E-01	**3.981E-01**
	**Std**	3.542E-13	1.129E-16	2.994E-05	2.225E-03	4.932E-04	2.548E-04	7.014E-04	2.599E-04
	*t***-values**	4.870E+00	4.870E+00	4.719E+00	−**4.369E+00**	1.373E+00	2.445E+00	−***6.332E-01***	
**F18**	**Mean**	***3.000E+00***	***3.000E+00***	***3.000E+00***	***3.000E+00***	***3.000E+00***	***3.000E+00***	***3.002E+00***	***3.000E+00***
	**Std**	2.176E-11	4.936E-15	2.155E-04	2.367E-04	6.735E-05	1.578E-04	2.204E-03	2.042E-04
	*t***-values**	5.105E+00	5.105E+00	2.362E+00	1.345E+00	3.469E+00	1.623E+00	−**4.590E+00**	
**F19**	**Mean**	−3.005E-01	−3.863E+00	−3.857E+00	−3.854E+00	−3.856E+00	−3.855E+00	−3.859E+00	−**3.860E+00**
	**Std**	0	2.710E-15	1.265E-02	2.246E-03	2.233E-03	1.396E-03	3.355E-03	2.636E-03
	*t***-values**	−**6.103E+03**	7.841E+00	−***7.052E-01***	−**5.772E+00**	−**3.793E+00**	−**5.030E+00**	***3.875E-01***	
**F20**	**Mean**	−2.396E+00	−3.274E+00	−3.226E+00	−2.853E+00	−2.820E+00	−2.999E+00	−3.178E+00	−**3.303E+00**
	**Std**	3.080E-01	5.924E-02	1.037E-01	3.779E-01	5.986E-01	2.991E-01	1.899E-01	1.761E-02
	*t***-values**	−**1.611E+01**	−**2.542E+00**	−**4.003E+00**	−**6.514E+00**	−**4.414E+00**	−**5.557E+00**	−**3.606E+00**	
**F21**	**Mean**	−8.806E+00	−5.131E+00	−8.229E+00	−2.804E+00	−2.724E+00	−2.573E+00	−7.055E+00	−**9.125E+00**
	**Std**	2.272E+00	2.780E+00	2.583E+00	1.664E+00	3.836E+00	3.679E+00	2.107E+00	6.835E-01
	*t***-values**	−***7.372E-01***	−**7.642E+00**	−**1.838E+00**	−**1.925E+01**	−**8.998E+00**	−**9.592E+00**	−**5.119E+00**	
**F22**	**Mean**	−8.635E+00	−7.421E+00	−7.601E+00	−3.209E+00	−7.160E+00	−6.161E+00	−6.375E+00	−**9.314E+00**
	**Std**	3.037E+00	3.346E+00	3.048E+00	1.756E+00	4.099E+00	4.659E+00	2.411E+00	5.378E-01
	*t***-values**	−***1.206E+00***	−**3.059E+00**	−**3.031E+00**	−**1.821E+01**	−**2.854E+00**	−**3.682E+00**	−**6.518E+00**	
**F23**	**Mean**	−9.055E+00	−8.556E+00	−6.353E+00	−4.123E+00	−7.482E+00	−7.641E+00	−5.651E+00	−**9.309E+00**
	**Std**	3.030E+00	3.142E+00	3.407E+00	1.750E+00	4.094E+00	3.877E+00	3.040E+00	6.429E-01
	*t***-values**	−***4.489E-01***	−***1.286E+00***	−**4.669E+00**	−**1.524E+01**	−**2.415E+00**	−**2.325E+00**	−**6.447E+00**	
**F24**	**Mean**	1.305E+07	1.703E+12	2.540E+10	7.830E+09	2.736E+08	2.908E+08	5.296E+04	**5.000E+04**
	**Std**	1.981E+07	1.145E+12	3.400E+10	1.070E+10	9.095E+08	4.846E+08	8.002E+03	5.554E+03
	*t***-values**	−**3.596E+00**	−**8.150E+00**	−**4.091E+00**	−**4.007E+00**	−***1.647E+00***	−**3.287E+00**	−***1.667E+00***	
**F25**	**Mean**	**1.734E+01**	1.449E+04	1.735E+01	1.749E+01	1.736E+01	1.740E+01	1.740E+01	1.738E+01
	**Std**	0	4.201E+03	1.134E-02	7.024E-02	6.212E-02	1.084E-01	5.558E-02	1.668E-02
	*t***-values**	1.239E+01	−**1.887E+01**	7.521E+00	−**8.110E+00**	***1.506E+00***	−***1.029E+00***	−***1.395E+00***	
**F26**	**Mean**	***1.270E+01***	***1.270E+01***	***1.270E+01***	***1.270E+01***	***1.270E+01***	***1.270E+01***	***1.270E+01***	***1.270E+01***
	**Std**	9.034E-15	9.034E-15	2.846E-06	9.209E-05	2.759E-04	4.556E-04	1.104E-05	6.065E-06
	*t***-values**	4.202E+00	4.202E+00	2.853E+00	−**6.355E+00**	−***9.907E-01***	−***9.988E-01***	−***9.066E-01***	
**F27**	**Mean**	**8.615E+00**	1.687E+01	3.871E+02	1.768E+03	7.421E+02	5.106E+02	8.032E+02	6.099E+02
	**Std**	2.937E+00	6.713E+00	2.315E+02	8.481E+02	9.160E+02	6.177E+02	5.973E+02	4.111E+02
	*t***-values**	8.012E+00	7.901E+00	2.587E+00	−**6.729E+00**	−***7.212E-01***	***7.332E-01***	−***1.460E+00***	
**F28**	**Mean**	**1.043E+00**	1.170E+00	1.994E+00	2.220E+00	1.936E+00	1.677E+00	2.382E+00	2.292E+00
	**Std**	2.676E-02	1.563E-01	3.850E-01	1.019E-01	2.995E-01	2.124E-01	5.014E-01	4.078E-01
	*t***-values**	1.674E+01	1.407E+01	2.911E+00	***9.320E-01***	3.848E+00	7.325E+00	−***7.633E-01***	
**F29**	**Mean**	9.906E+00	1.005E+01	9.908E+00	1.111E+01	1.105E+01	1.099E+01	1.095E+01	**9.767E+00**
	**Std**	1.856E+00	1.043E+00	8.051E-01	6.115E-01	7.709E-01	7.221E-01	6.059E-01	1.098E+00
	*t***-values**	−***3.518E-01***	−***1.010E+00***	−***5.651E-01***	−**5.865E+00**	−**5.248E+00**	−**5.098E+00**	−**5.185E+00**	
**F30**	**Mean**	**6.441E+01**	3.476E+02	6.691E+02	7.824E+02	5.238E+02	4.262E+02	7.350E+02	6.056E+02
	**Std**	9.273E+01	1.222E+02	2.756E+02	1.950E+02	1.396E+02	1.808E+02	2.253E+02	1.800E+02
	*t***-values**	1.464E+01	6.497E+00	−***1.056E+00***	−**3.648E+00**	1.969E+00	3.854E+00	−**2.457E+00**	
**F31**	**Mean**	5.815E+00	5.802E+00	6.130E+00	6.091E+00	6.299E+00	6.407E+00	6.367E+00	**5.797E+00**
	**Std**	5.042E-01	5.147E-01	5.582E-01	4.716E-01	4.345E-01	4.128E-01	3.658E-01	3.661E-01
	*t***-values**	−***1.509E-01***	−***3.797E-02***	−**2.727E+00**	−**2.693E+00**	−**4.831E+00**	−**6.047E+00**	−**6.032E+00**	
**F32**	**Mean**	2.483E+00	**2.374E+00**	4.773E+00	1.407E+02	1.737E+02	1.633E+01	4.231E+01	2.094E+01
	**Std**	1.066E-01	2.027E-02	8.403E-01	1.071E+02	2.451E+02	5.251E+01	7.767E+01	4.468E+01
	*t***-values**	2.262E+00	2.276E+00	1.981E+00	−**5.654E+00**	−**3.360E+00**	***3.659E-01***	−***1.306E+00***	
**F33**	**Mean**	***2.022E+01***	2.036E+01	2.035E+01	2.048E+01	2.053E+01	2.053E+01	2.048E+01	***2.022E+01***
	**Std**	1.033E-01	1.755E-01	1.388E-01	1.020E-01	8.130E-02	6.480E-02	8.093E-02	1.177E-01
	*t***-values**	−***4.197E-02***	−**3.700E+00**	−**3.948E+00**	−**9.224E+00**	−**1.206E+01**	−**1.256E+01**	−**9.915E+00**	

**w/t/l**		11/9/13	16/4/13	15/5/13	28/3/2	21/4/8	21/4/8	14/15/4	

**Figure 14:**
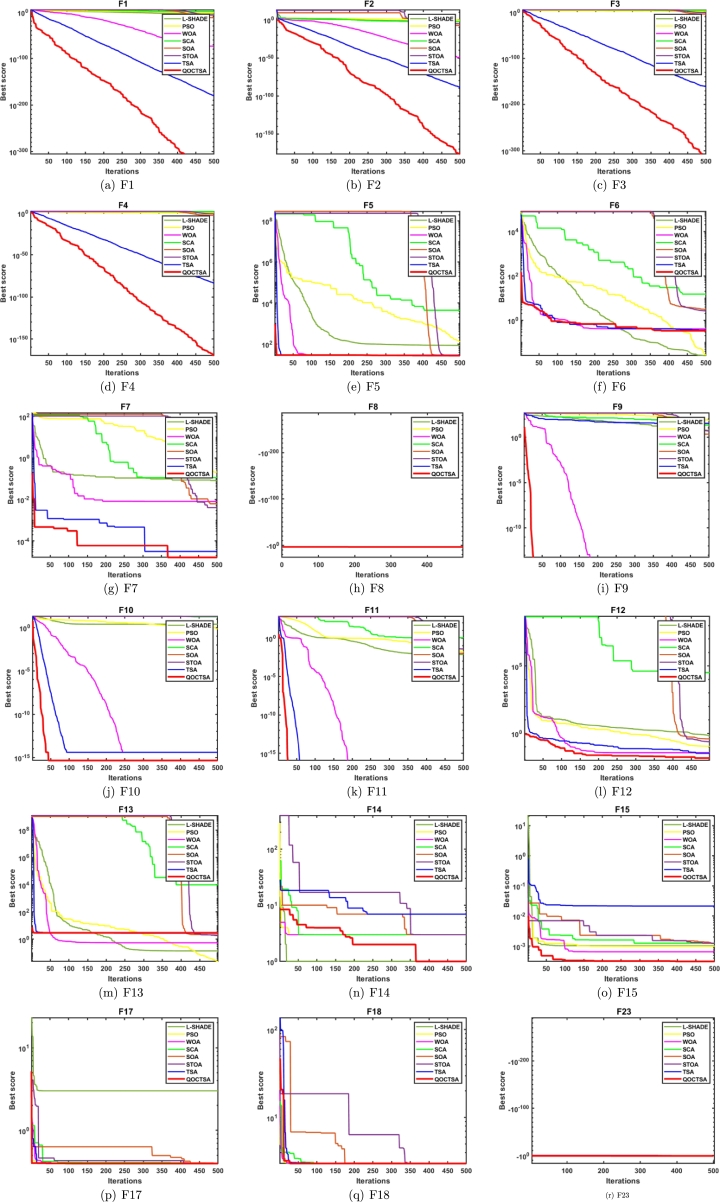
Convergence graphs of the QOCTSA on CEC2005 test functions.

**Figure 15:**
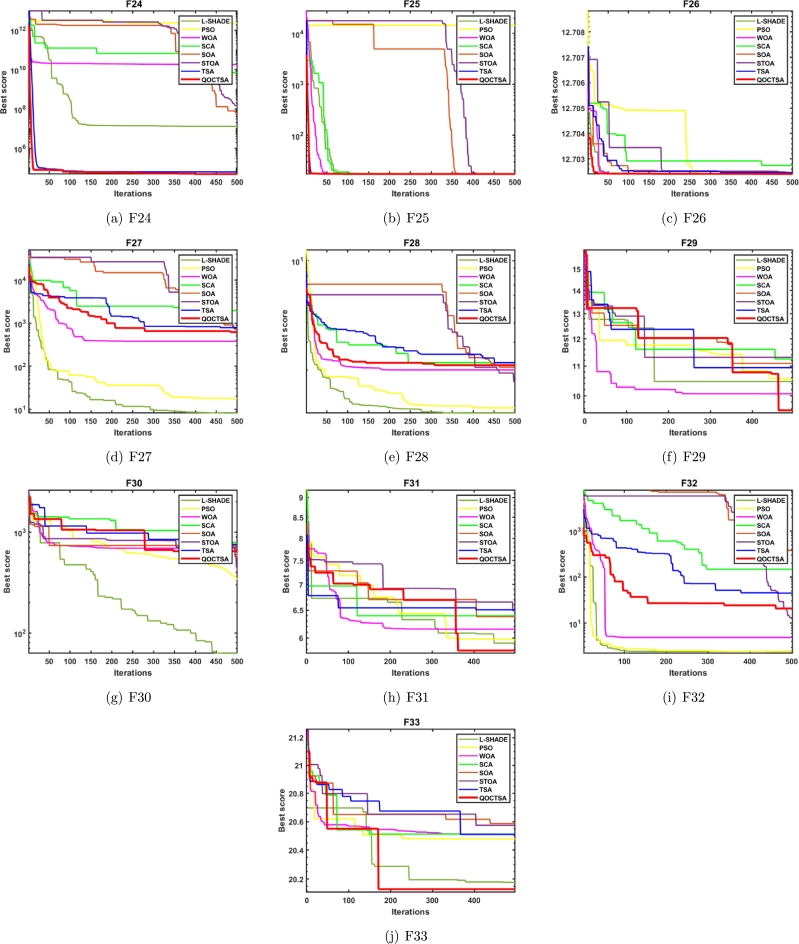
Convergence graphs of the QOCTSA on CEC2019 test functions.

The convergence analysis examines the optimization performance and provides a graphical depiction of the suggested QOCTSA method. Figure 14, Figure 15 outline the average convergence curves of CEC2005 and CEC2019 test functions, respectively. The curves depicted in Figs. 14(a) to 14(r) show that QOCTSA achieves rapid convergence and a maximum level of accuracy on unimodal test functions. In particular, the QOCTSA quickly converges to the optimal point for the function F1, while the other functions exhibit either mediocre convergence or an inability to converge towards the optimal point. In the multimodal functions, QOCTSA still maintains the highest rate of convergence for all test functions. Furthermore, QOCTSA successfully determines the global optimum in both F9 and F11. Although the suggested method is unable to find the global best for other functions, QOCTSA outperformed the other algorithms. The efficiency of the QOCTSA method for fixed-dimensional multimodal functions also demonstrates rapid convergence and strong convergence accuracy. The convergence curves of the CEC2019 test functions in Figs. 15(a) to 15(j) reveal that the QOCTSA exhibits a higher rate of convergence for all the functions except F27, F28, F30, and F32.

**Figure 16:**
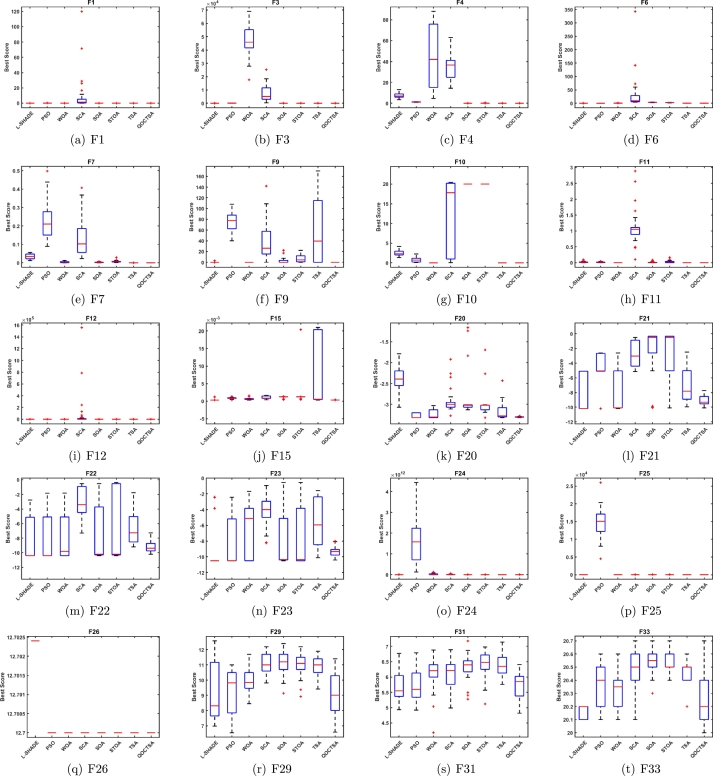
Boxplots of QOCTSA and other competing algorithms on some CEC2005 and CEC2019 test functions.

In addition, robustness is a crucial metric for assessing the algorithm's optimization performance. If the algorithm's robustness is poor, it'll be challenging to converge to the optimal region in each iteration. If not, even with its significant adaptability, it might reach a satisfactory degree of convergence to the optimal region in each iteration. Since MHAs primarily rely on randomness, numerous runs may result in varying fitness values. In order to have a clear idea of the nature and distribution of the generated solutions, this section also discusses the visualization of boxplot analysis of the proposed QOCTSA. In general, a boxplot analysis provides insight into the characteristics of distributed data. Specifically, boxplot displays a five-tuple description of the data: the $1^{st}$ quartile, the median or $2^{nd}$ quartile, the $3^{rd}$ quartile, and the minimum and maximum values. The minimum and maximum values of the algorithm are represented by the straight line that extends from the box's edge. Thus, the boxplot charts of the CEC2005 and CEC2019 test functions for the QOCTSA and other competing algorithms are depicted in Fig. 16 after 30 independent executions. Upon closer investigation, the boxplots presented in Figs. 16(a) to 16(t) reveal that the majority of functions' boxplots are more compact than other algorithms. A compact boxplot signifies strong data distribution and precision. Hence, the proposed QOCTSA outperforms other competing algorithms by achieving strong data distribution, demonstrating the robustness and reliability of QOCTSA in boxplot analysis.

In addition, the *t*-test [102] and non-parametric pair-wise Wilcoxon rank-sum test [103] are conducted to evaluate the statistical significance of the simulation outcomes obtained from the proposed QOCTSA method in comparison to the simulation outcomes of the other algorithms. Table 11 presents the results of the *t*-test at the 5% significance level for the standard CEC2005 and CEC2019 test functions. If the corresponding *t*-values are in bold text, QOCTSA performs better than the other competing algorithms, whereas the bold italics indicate a tie. Moreover, it should be noted that the final row of Table 11 is labelled w/t/l, which represents the win, tie, and lose totals of the suggested QOCTSA technique. Hence, the *t*-values in Table 11 reveal that the effectiveness of the QOCTSA approach has increased significantly in several cases. Furthermore, a non-parametric pair-wise Wilcoxon rank-sum test can determine the significant difference between two algorithms. The *p*-values generated by performing the Wilcoxon rank-sum test at a significance level of 5% are illustrated in Table 12. The *p*-values below 0.05 indicate a statistically significant difference between the two algorithms. In Table 12, the symbols ‘+’ and ‘-’ in the H column indicate the significance and insignificance differences between the QOCTSA and other algorithms. Here, the term ‘NA’ means there is no statistical difference between the algorithms. The above-mentioned table demonstrates that nearly all *p*-values are below 0.05 and concludes that the suggested QOCTSA technique outperformed the other compared algorithms. In general, the performance of the QOCTSA in comparative tests demonstrates the strong exploitation and exploration abilities and proves the effectiveness of the two introduced mechanisms.

**Table 12:** Outcomes of the Wilcoxon rank sum test on CEC2005 and CEC2019 test functions (the best outcomes are highlighted in bold text).

**Func.**	**QOCTSA vs L-SHADE**		**QOCTSA vs PSO**		**QOCTSA vs WOA**		**QOCTSA vs SCA**		**QOCTSA vs SOA**		**QOCTSA vs STOA**		**QOCTSA vs TSA**	
	*p*-values	H	*p*-values	H	*p*-values	H	*p*-values	H	*p*-values	H	*p*-values	H	*p*-values	H
**F1**	1.776E-06	+	1.775E-06	+	1.777E-06	+	1.778E-06	+	1.779E-06	+	1.777E-06	+	1.776E-06	+
**F2**	1.774E-06	+	1.776E-06	+	1.775E-06	+	1.775E-06	+	1.778E-06	+	1.776E-06	+	1.778E-06	+
**F3**	1.778E-06	+	1.776E-06	+	1.775E-06	+	1.777E-06	+	1.775E-06	+	1.778E-06	+	1.779E-06	+
**F4**	1.778E-06	+	1.779E-06	+	1.777E-06	+	1.776E-06	+	1.775E-06	+	1.777E-06	+	1.778E-06	+
**F5**	8.312E-02	-	1.779E-06	+	7.292E-04	+	1.779E-06	+	1.392E-05	+	8.671E-06	+	3.007E-03	+
**F6**	1.778E-06	+	1.775E-06	+	1.863E-01	-	1.777E-06	+	1.778E-06	+	1.777E-06	+	6.325E-01	-
**F7**	1.778E-06	+	1.777E-06	+	1.971E-06	+	1.775E-06	+	1.776E-06	+	1.778E-06	+	2.598E-02	+
**F8**	1.778E-06	+	7.878E-06	+	1.778E-06	+	1.121E-01	-	2.952E-06	+	7.155E-06	+	1.373E-01	-
**F9**	1.776E-06	+	1.778E-06	+	NA	-	1.777E-06	+	1.776E-06	+	1.776E-06	+	9.207E-05	+
**F10**	1.764E-06	+	1.779E-06	+	2.589E-05	+	1.779E-06	+	1.779E-06	+	1.779E-06	+	4.467E-08	+
**F11**	1.770E-06	+	1.779E-06	+	NA	-	1.779E-06	+	1.779E-06	+	1.779E-06	+	NA	-
**F12**	1.031E-01	-	2.812E-03	+	8.653E-01	-	1.777E-06	+	1.776E-06	+	1.778E-06	+	1.621E-03	+
**F13**	1.777E-06	+	1.776E-06	+	1.778E-06	+	1.778E-06	+	2.952E-06	+	3.604E-06	+	3.111E-01	-
**F14**	1.042E-06	+	1.323E-02	+	1.372E-03	+	4.439E-03	+	2.139E-03	+	2.646E-04	+	1.258E-02	+
**F15**	3.183E-05	+	1.971E-06	+	4.393E-06	+	1.777E-06	+	1.778E-06	+	1.778E-06	+	3.262E-06	+
**F16**	1.777E-06	+	1.778E-06	+	1.776E-06	+	9.111E-05	+	3.183E-05	+	7.043E-05	+	6.770E-01	-
**F17**	1.778E-06	+	1.778E-06	+	5.346E-06	+	1.268E-05	+	6.640E-02	-	1.121E-01	-	5.338E-01	-
**F18**	1.779E-06	+	1.779E-06	+	2.270E-04	+	9.469E-02	-	5.757E-03	+	2.077E-01	-	2.019E-05	+
**F19**	1.779E-06	+	1.779E-06	+	4.940E-01	-	6.459E-05	+	3.918E-03	+	3.958E-04	+	8.170E-01	-
**F20**	1.779E-06	+	2.390E-01	-	3.729E-02	+	1.778E-06	+	1.777E-06	+	2.414E-06	+	3.660E-04	+
**F21**	6.621E-01	-	1.529E-05	+	3.962E-01	-	1.779E-06	+	1.154E-05	+	7.155E-06	+	9.111E-05	+
**F22**	6.770E-01	-	4.774E-02	+	4.774E-02	+	1.779E-06	+	2.077E-01	-	4.774E-02	+	4.847E-06	+
**F23**	1.666E-01	-	9.795E-01	-	7.857E-04	+	1.779E-06	+	3.520E-01	-	3.848E-01	-	2.952E-06	+
**F24**	1.778E-06	+	1.777E-06	+	1.776E-06	+	1.775E-06	+	1.971E-06	+	1.778E-06	+	1.863E-01	-
**F25**	1.777E-06	+	1.776E-06	+	2.670E-06	+	1.778E-06	+	3.183E-05	+	1.666E-01	-	9.882E-02	-
**F26**	1.778E-06	+	1.776E-06	+	5.890E-06	+	1.777E-06	+	2.004E-01	-	5.777E-02	-	8.977E-01	-
**F27**	1.779E-06	+	1.779E-06	+	5.010E-02	-	2.952E-06	+	8.977E-01	-	3.111E-01	-	1.796E-01	-
**F28**	1.779E-06	+	1.779E-06	+	1.865E-03	+	7.074E-01	-	4.622E-04	+	3.604E-06	+	2.230E-01	-
**F29**	6.621E-01	-	1.863E-01	-	6.621E-01	-	4.968E-05	+	1.174E-04	+	1.678E-05	+	1.678E-05	+
**F30**	1.779E-06	+	8.671E-06	+	5.893E-01	-	1.739E-03	+	3.921E-02	+	2.888E-04	+	4.121E-02	+
**F31**	7.852E-01	-	8.653E-01	-	4.758E-03	+	2.739E-02	+	7.857E-04	+	7.677E-05	+	9.111E-05	+
**F32**	1.779E-06	+	1.779E-06	+	3.980E-06	+	1.392E-05	+	1.031E-01	-	1.192E-02	+	3.415E-01	-
**F33**	9.795E-01	-	1.192E-02	+	3.958E-04	+	5.346E-06	+	1.779E-06	+	1.779E-06	+	2.181E-06	+

### 4.5 Ablation experiment

The QOCTSA is an ameliorated TSA variant developed by a novel incorporation of QOBL, CLS, and the standard TSA, resulting in superior algorithm convergence and endurance. The research studies suggest that the cooperation between the core of TSA and the optimization aspects can reveal the versatility of the proposed QOCTSA in handling various task kinds. As a result, this subsection employs an ablation experiment to demonstrate more vividly the influence of proposed strategies (QOBL and CLS) on TSA with QOCTSA in further detail.

**Figure 17:**
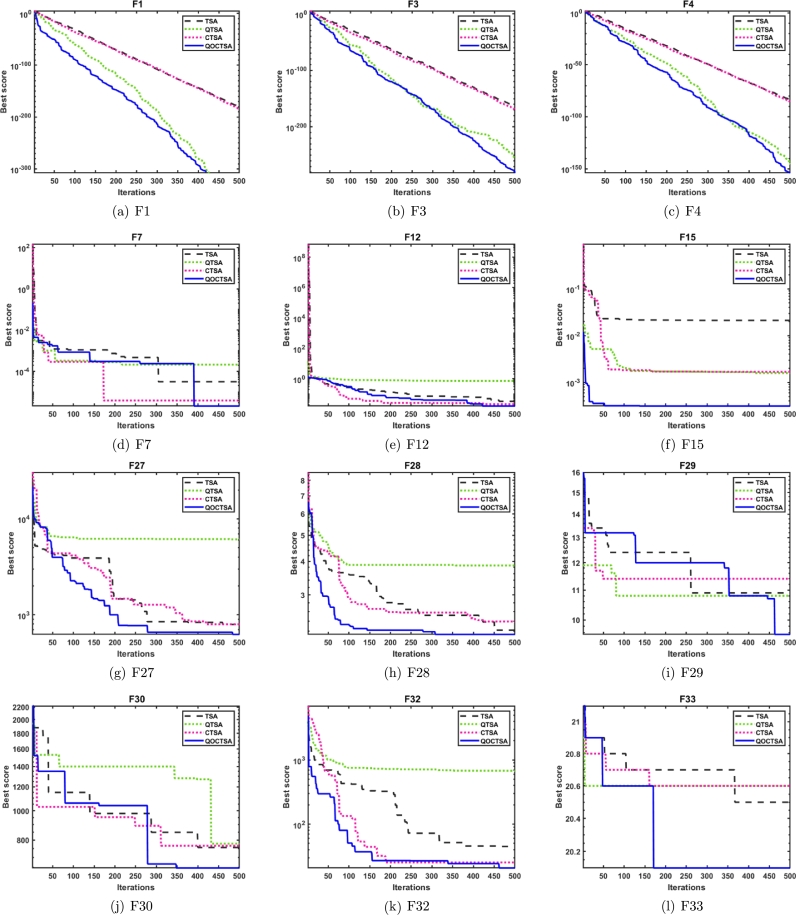
Convergence curves of the ablation experiment on some CEC2005 and CEC2019 test functions.

The pairing information of QOBL and CLS strategies with TSA is displayed in Table 13. The symbols “✓” and “×” signify the employment and non-use of the corresponding strategy, respectively (Note: QTSA (Quasi-opposition based TSA) and CTSA (Chaotic based TSA)). The comparative findings of the ablation experiment on a diverse set of thirty-three test functions are listed in Table 14. It is worth mentioning that every algorithm used in the ablation experiments was independently performed a total of 30 times, employing 30 search agents and a maximum of 500 iterations. According to the statistical findings in Table 14, the range of ‘Mean’ among the four algorithms reveals that the proposed QOCTSA outperformed the other algorithms on the majority of functions. Furthermore, the spectrum of ‘Std’ confirms that the performance of QOCTSA is more stable than that of QTSA, CTSA, and TSA. In order to further investigate the influence of QOBL and CLS strategies on TSA, Fig. 17 depicts the graphical representation of QOCTSA with the other three variants on several test functions. The convergence curves in Figs. 17(a) to 17(l) highlight that QOCTSA has excellent convergence performance and exhibits that QOBL and CLS strategies can assist QOCTSA in exploring and exploiting the potential regions in most cases. In summary, it can be concluded that QTSA, CTSA, and QOCTSA all surpass TSA, specifically QOCTSA performing more efficiently than QTSA and CTSA.

**Table 13:** The TSA variants of the ablation experiment.

**Algorithms**	**QOBL**	**CLS**
QOCTSA	✓	✓
QTSA	✓	×
CTSA	×	✓
TSA	×	×

**Table 14:** Comparison outcomes of the ablation experiment on CEC2005 and CEC2019 test functions.

	**F1**		**F2**		**F3**		**F4**		**F5**	
	Mean	Std	Mean	Std	Mean	Std	Mean	Std	Mean	Std

TSA	5.301E-180	0.000E+00	4.791E-85	2.624E-84	9.718E-160	0.000E+00	3.090E-84	1.427E-83	2.804E+01	8.960E-01
QTSA	0.000E+00	0.000E+00	8.469E-162	0.000E+00	1.300E-248	0.000E+00	2.557E-142	1.400E-141	2.823E+01	7.754E-01
CTSA	2.512E-187	0.000E+00	2.403E-94	4.927E-94	1.337E-169	0.000E+00	4.747E-86	7.727E-86	2.799E+01	8.248E-01
QOCTSA	**0.000E+00**	**0.000E+00**	**3.595E-163**	**0.000E+00**	**1.039E-280**	**0.000E+00**	**6.947E-151**	**3.805E-150**	**2.744E+01**	**7.257E-01**

	**F6**		**F7**		**F8**		**F9**		**F10**	

	Mean	Std	Mean	Std	Mean	Std	Mean	Std	Mean	Std

TSA	3.507E-01	7.660E-02	1.025E-04	1.088E-04	−**4.130E+03**	**3.551E+02**	5.552E+01	5.809E+01	3.997E-15	2.407E-30
QTSA	2.690E+00	9.838E-01	9.543E-05	7.627E-05	−3.814E+03	8.010E+02	***0.000E+00***	***0.000E+00***	***4.441E-16***	***3.009E-31***
CTSA	3.496E-01	7.730E-02	7.194E-05	6.513E-05	−3.965E+03	4.686E+02	***0.000E+00***	***0.000E+00***	***4.441E-16***	***3.009E-31***
QOCTSA	**3.161E-01**	**6.621E-02**	**5.608E-05**	**4.291E-05**	−4.002E+03	6.437E+02	***0.000E+00***	***0.000E+00***	***4.441E-16***	***3.009E-31***

	**F11**		**F12**		**F13**		**F14**		**F15**	

	Mean	Std	Mean	Std	Mean	Std	Mean	Std	Mean	Std

TSA	***0.000E+00***	***0.000E+00***	3.813E-02	5.445E-02	**2.798E+00**	2.227E-01	**4.249E+00**	4.906E+00	7.210E-03	9.631E-03
QTSA	***0.000E+00***	***0.000E+00***	2.643E-01	2.507E-01	2.955E+00	1.531E-01	8.861E+00	**4.734E+00**	5.046E-03	8.657E-03
CTSA	***0.000E+00***	***0.000E+00***	2.119E-02	6.201E-03	2.912E+00	**1.272E-01**	4.573E+00	5.397E+00	3.801E-03	7.661E-03
QOCTSA	***0.000E+00***	***0.000E+00***	**2.088E-02**	**5.493E-03**	2.855E+00	2.584E-01	8.101E+00	5.705E+00	**3.229E-04**	**1.775E-05**

	**F16**		**F17**		**F18**		**F19**		**F20**	

	Mean	Std	Mean	Std	Mean	Std	Mean	Std	Mean	Std

TSA	−**1.032E+00**	**1.174E-05**	3.985E-01	7.014E-04	3.002E+00	2.204E-03	−3.859E+00	3.355E-03	−3.178E+00	1.899E-01
QTSA	−1.022E+00	1.475E-02	3.992E-01	2.927E-03	8.401E+00	1.099E+01	−**3.861E+00**	**2.458E-03**	−3.267E+00	6.811E-02
CTSA	−1.032E+00	1.796E-05	3.989E-01	1.373E-03	5.701E+00	1.479E+01	−3.857E+00	3.395E-03	−3.055E+00	3.145E-01
QOCTSA	−1.032E+00	1.627E-05	**3.983E-01**	**4.539E-04**	**3.001E+00**	**1.343E-03**	−3.858E+00	3.370E-03	−**3.307E+00**	**7.504E-03**

	**F21**		**F22**		**F23**		**F24**		**F25**	

	Mean	Std	Mean	Std	Mean	Std	Mean	Std	Mean	Std

TSA	−7.055E+00	2.107E+00	−6.375E+00	2.411E+00	−5.651E+00	3.040E+00	5.296E+04	8.002E+03	1.738E+01	1.157E-02
QTSA	−8.958E+00	1.127E+00	−8.863E+00	1.569E+00	−8.942E+00	1.544E+00	7.707E+04	2.764E+04	1.797E+01	4.765E-01
CTSA	−7.086E+00	1.862E+00	−6.952E+00	2.528E+00	−5.727E+00	2.760E+00	5.024E+04	6.292E+03	1.739E+01	4.890E-02
QOCTSA	−**9.090E+00**	**6.269E-01**	−**9.221E+00**	**7.355E-01**	−**9.304E+00**	**5.828E-01**	**4.903E+04**	**5.984E+03**	**1.737E+01**	**9.538E-03**

	**F26**		**F27**		**F28**		**F29**		**F30**	

	Mean	Std	Mean	Std	Mean	Std	Mean	Std	Mean	Std

TSA	1.270E+01	**9.895E-06**	8.032E+02	5.973E+02	2.382E+00	5.014E-01	1.095E+01	**6.059E-01**	7.350E+02	2.253E+02
QTSA	1.270E+01	8.170E-04	5.956E+03	2.613E+03	3.483E+00	9.003E-01	1.080E+01	6.310E-01	7.390E+02	2.598E+02
CTSA	1.270E+01	2.236E-05	7.854E+02	7.281E+02	2.215E+00	2.857E-01	1.112E+01	6.576E-01	7.299E+02	2.411E+02
QOCTSA	**1.270E+01**	1.324E-05	**5.701E+02**	**4.384E+02**	**1.904E+00**	**1.147E-01**	**9.091E+00**	1.354E+00	**4.149E+02**	**1.161E+02**

	**F31**		**F32**		**F33**					

	Mean	Std	Mean	Std	Mean	Std				

TSA	6.367E+00	3.658E-01	4.231E+01	7.767E+01	2.048E+01	**8.093E-02**				
QTSA	6.446E+00	**3.337E-01**	7.376E+02	5.229E+02	2.042E+01	1.320E-01				
CTSA	6.401E+00	3.700E-01	2.760E+01	**5.513E+01**	2.047E+01	8.734E-02				
QOCTSA	**5.742E+00**	4.553E-01	**2.631E+01**	7.106E+01	**2.013E+01**	1.024E-01

It is worth noting that, according to the CTSA findings, the chaotic sequence not only boost but rather widened the performance of the algorithm. The reason for this is that chaos is incredibly unpredictable, so to find a better individual, the initial set of individuals requires a stronger search mechanism. But from the findings of QTSA, by adopting the QOBL strategy in population initialization and generation jumping, the quasi-opposition individuals significantly enhance the spatial searchability of the algorithm. This signifies that QOBL assists in exploring the search area more intensively and enhances diversification, whereas chaotic functions emphasize the most promising regions of the search area and improve intensification, further demonstrating that the QOCTSA with both incorporated strategies are the best-improved version in this study. The ablation experiment not merely confirms the success of the proposed approach but also discloses the unique implications of various improved strategies, offering insightful information for the development and design of new algorithms in the future.

## 5 Comparison of QOCTSA in real-world engineering design problems

In this section, four real-world engineering design problems, namely, tension or compression spring design, pressure vessel design, welded beam design, and three-bar truss design problems, are utilized to further analyse the performance of the proposed QOCTSA technique. The description of these constrained engineering design problems with optimal values is provided in Table 15. And the outcomes of the QOCTSA are tested and statistically analyzed in comparison to those of the standard TSA and several other MHAs, including PSO, WOA, SCA, SOA, STOA, and AOA.

**Table 15:** Description of real-world engineering design problems (D is the dimension of the problem, *g* and *h* are number of inequality and equality constraints).

**Design problems**	**Objectives**	**D**	***g***	***h***	**Range**	$f_{min}$
Compression spring [104]	To optimize the weight of tension or compression spring	3	3	0	[0.05,0.25,2; 2,1.3,15]	1.26653E-02
Pressure vessel [105]	To reduce the vessel cost of cylindrical pressure	4	4	0	[0,0,10,10; 99,99,200,200]	5.88530E+03
Welded beam [105]	To minimize the fabrication cost of welded beam	4	5	0	[0.1,0.1,0.1,0.1; 2,10,10,2]	1.67021E+00
Three-bar truss [106]	To optimize the volume of the loaded truss structure	2	3	0	[0,0; 1,1]	2.63895E+02

### 5.1 Tension or compression spring design problem

**Figure 18:**
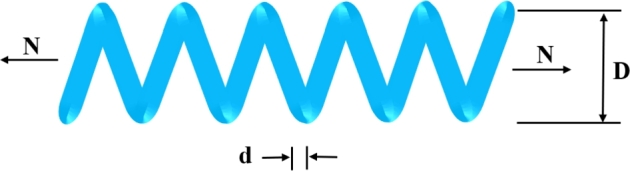
Tension or compression spring design.

The primary goal of this engineering problem is to optimize the spring's weight. The parameters employed for formulating this problem are the diameter of the wire (*d*), the mean coil diameter (*D*), and the number of active coils (*N*). Fig. 18 depicts the simplified model of the spring. Also, the objective function of this design is represented numerically in Eq. (16), subjected to the constraints specified in Eqs. (17) to (20).

Suppose, $\overset{\to }{x}=[x_{1},x_{2},x_{3}]=[d\;D\;N]$

(16) $$Minimize\;f(\overset{\to }{x})=(x_{3}+2)x_{2}x_{1}^{2}$$

subject to

(17) $$g_{1}(\overset{\to }{x})=1-\frac{x_{2}^{3}x_{3}}{71785x_{1}^{4}}\leq 0,$$

(18) $$g_{2}(\overset{\to }{x})=\frac{4x_{2}^{2}-x_{1}x_{2}}{12566(x_{2}x_{1}^{3}-x_{1}^{4})}+\frac{1}{5108x_{1}^{2}}\leq 0,$$

(19) $$g_{3}(\overset{\to }{x})=1-\frac{140.45x_{1}}{x_{2}^{2}x_{3}}\leq 0,$$

(20) $$g_{4}(\overset{\to }{x})=\frac{x_{1}+x_{2}}{1.5}-1\leq 0.$$

Variables range:

$$0.05\leq x_{1}\leq 2.00,0.25\leq x_{2}\leq 1.30,2.00\leq x_{3}\leq 15.0.$$

**Figure 19:**
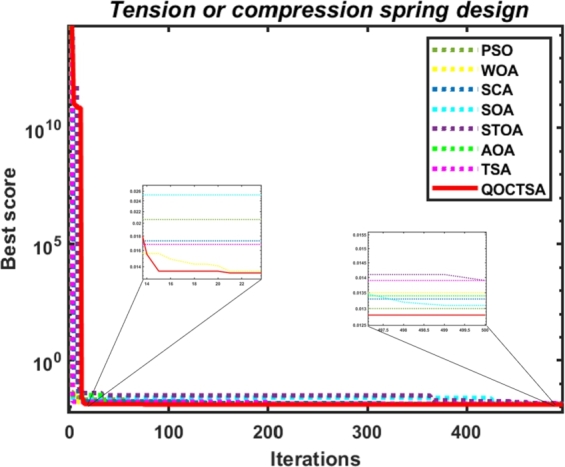
Convergence curve of tension or compression spring design.

Table 16, Table 17 compare the best outcomes of the proposed QOCTSA with seven other competing MHAs, i.e., PSO, WOA, SCA, SOA, STOA, AOA, and TSA. From Table 16, it is evident that the outcomes of the QOCTSA have reduced the weight of the spring under the optimal solution [$x_{1}$, $x_{2}$, $x_{3}$]=[7.985E-01, 4.603E-01, 4.117E+01, 1.922E+02] by satisfying the constraints and the corresponding weight function value $f(\overset{\to }{(}x)$= 6.227E+03 in comparison to other reported algorithms. Table 17 records the minimum value (Best), mean of the minimum value (Mean), standard deviation (Std), maximum value (Worst), and the *p*-values of the objective function for all algorithms after 30 independent runs. This table also reveals that the QOCTSA outperformed in all measures and obtained the least weight of the spring, followed by PSO. The p-value between QOCTSA and each algorithm presented in this table indicates that the outcomes of QOCTSA are significantly different from those of other competing algorithms. Further evidence for this can be observed in the zoomed region of convergence graph in Fig. 19, which shows that the QOCTSA converges rapidly towards the close-to-optimal solution after initial iterations. This convergence depicts the relationship between the mean function value and the iteration number of the QOCTSA and other competing algorithms compiled over 500 iterations. Thus, the experimental findings of this engineering design demonstrate the efficacy and reliability of the proposed QOCTSA in dealing with the compression spring problem.

**Table 16:** Best outcomes of the competing algorithms for solving the tension or compression spring design problem.

**Algorithms**	**Optimal values of variables**			**Minimum weight**
	*d*	*D*	*N*	
**PSO**	5.512E-02	4.450E-01	7.519E+00	1.287E-02
**WOA**	5.685E-02	4.942E-01	6.215E+00	1.312E-02
**SCA**	5.452E-02	4.251E-01	8.301E+00	1.302E-02
**SOA**	5.518E-02	4.466E-01	7.485E+00	1.290E-02
**STOA**	5.640E-02	4.804E-01	6.664E+00	1.324E-02
**AOA**	5.000E-02	3.139E-01	1.500E+01	1.334E-02
**TSA**	5.759E-02	5.092E-01	6.153E+00	1.377E-02
**QOCTSA**	5.000E-02	3.168E-01	1.413E+01	**1.277E-02**

**Table 17:** Comparison outcomes of the tension or compression spring design problem.

**Algorithms**	**Best**	**Mean**	**Std**	**Worst**	*p***-values**
**PSO**	1.287E-02	1.329E-02	7.360E-04	1.567E-02	2.761E-02
**WOA**	1.312E-02	1.401E-02	1.106E-03	1.622E-02	2.182E-05
**SCA**	1.302E-02	1.313E-02	2.080E-04	1.398E-02	2.227E-03
**SOA**	1.290E-02	1.321E-02	7.616E-04	1.559E-02	2.023E-01
**STOA**	1.301E-02	1.311E-02	6.419E-04	1.560E-02	3.835E-01
**AOA**	1.334E-02	1.732E-02	7.440E-03	3.481E-02	6.536E-06
**TSA**	1.377E-02	1.388E-02	1.276E-03	1.856E-02	2.098E-05
**QOCTSA**	**1.277E-02**	**1.294E-02**	**1.808E-04**	1.357E-02

### 5.2 Pressure vessel design problem

**Figure 20:**
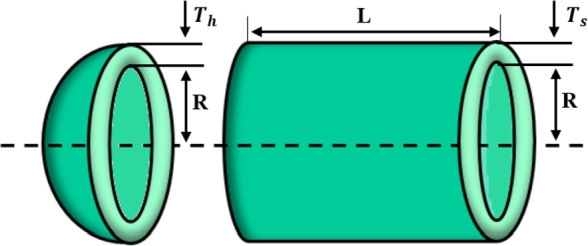
Pressure vessel design.

The main aim of this engineering design is to minimize the material, welding, and forming costs of a cylindrical pressure vessel. The schematic depiction of the pressure vessel is outlined in Fig. 20. The parameters that need to be optimized for this design are the thickness of the shell $\left(T_{s}\right)$, the thickness of the head $\left(T_{h}\right)$, the inner radius (*R*), and the length of the cylinder except the head (*L*). Furthermore, Eq. (21) provides a mathematical formulation of the objective function of this design with respect to the constraints outlined in Eqs. (22) to (25).

Suppose, $\overset{\to }{y}=[y_{1},y_{2},y_{3},y_{4}]=[T_{s}\;T_{h}\;R\;L]$

(21) $$Minimize\;f(\overset{\to }{y})=0.6224y_{1}y_{3}y_{4}+1.7781y_{2}y_{3}^{2}+3.1661y_{1}^{2}y_{4}+19.84y_{1}^{2}y_{3}$$

subject to

(22) $$g_{1}(\overset{\to }{y})=-y_{1}+0.0193y_{3}\leq 0,$$

(23) $$g_{2}(\overset{\to }{y})=-y_{2}+0.00954y_{3}\leq 0,$$

(24) $$g_{3}(\overset{\to }{y})=-\pi y_{3}^{2}y_{4}-\frac{4}{3}\pi y_{3}^{3}+1296000\leq 0,$$

(25) $$g_{4}(\overset{\to }{y})=y_{4}-240\leq 0.$$

Variables range:

$$0\leq y_{1}\leq 99,0\leq y_{2}\leq 99,10\leq y_{3}\leq 200,10\leq y_{4}\leq 200.$$

**Figure 21:**
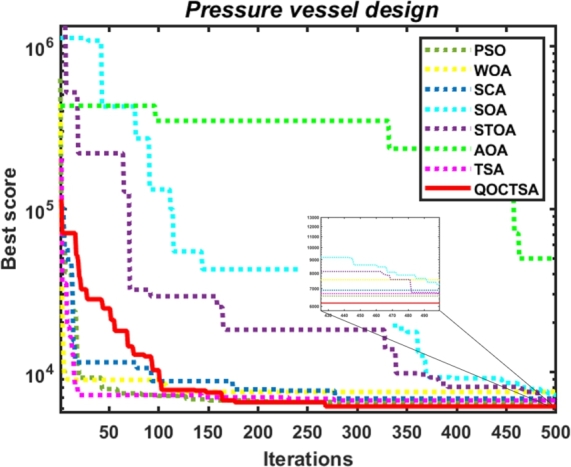
Convergence curve of pressure vessel design.

This engineering design is handled by employing the optimal outcomes of QOCTSA and other competing algorithms, with a maximum of 500 iterations and 30 search agents. These algorithms are conducted by 30 independent runs, and their outcomes are investigated using several metrics, which are reported in Table 18, Table 19. From Table 18, it is observed that the optimal value of QOCTSA is [$y_{1}$, $y_{2}$, $y_{3}$, $y_{4}$]=[7.985E-01, 4.603E-01, 4.117E+01, 1.922E+02] with the respective cost function value $f(\overset{\to }{(}y)$= 5.870E+03. Thus, the optimal cost of QOCTSA outperforms all other methods. Also, based on the information provided in Table 19, QOCTSA surpasses the other competing algorithms for all metrics. Further, Fig. 21 demonstrates the rapid convergence of QOCTSA against other algorithms for the pressure vessel design problem. Specifically, the intensified region of Fig. 21 demonstrates that QOCTSA has better and more significant convergence ability. In simple terms, the proposed QOCTSA performs splendidly in solving the pressure vessel problem.

**Table 18:** Best outcomes of the competing algorithms for solving the pressure vessel design problem.

**Algorithms**	**Optimal values of variables**				**Minimum cost**
	$T_{s}$	$T_{h}$	*R*	*L*	
**PSO**	1.065E+00	5.262E-01	5.516E+01	6.205E+01	6.577E+03
**WOA**	9.938E-01	6.232E-01	4.541E+01	1.395E+02	7.530E+03
**SCA**	9.373E-01	5.022E-01	4.791E+01	1.270E+02	6.787E+03
**SOA**	1.230E+00	5.911E-01	6.184E+01	2.590E+01	6.326E+03
**STOA**	7.873E-01	5.806E-01	4.037E+01	1.997E+02	6.522E+03
**AOA**	2.076E+00	1.770E+00	1.028E+02	7.111E+01	1.337E+04
**TSA**	1.026E+00	5.016E-01	5.175E+01	8.708E+01	6.637E+03
**QOCTSA**	7.985E-01	4.603E-01	4.117E+01	1.922E+02	**5.870E+03**

**Table 19:** Comparison outcomes of the pressure vessel design problem.

**Algorithms**	**Best**	**Mean**	**Std**	**Worst**	*p***-values**
**PSO**	6.577E+03	6.823E+03	2.732E+02	7.296E+03	2.868E-01
**WOA**	7.530E+03	1.141E+04	1.216E+04	7.446E+04	1.971E-06
**SCA**	6.787E+03	7.569E+03	8.662E+02	9.229E+03	2.948E-06
**SOA**	6.326E+03	6.781E+03	4.744E+02	7.436E+03	3.605E-02
**STOA**	6.522E+03	6.371E+03	4.662E+02	7.422E+03	6.107E-01
**AOA**	1.337E+04	4.493E+04	4.525E+04	2.009E+05	1.779E-06
**TSA**	6.637E+03	7.223E+03	5.854E+02	8.351E+03	3.977E-06
**QOCTSA**	**5.870E+03**	**6.264E+03**	**2.438E+02**	6.689E+03

### 5.3 Welded beam design problem

**Figure 22:**
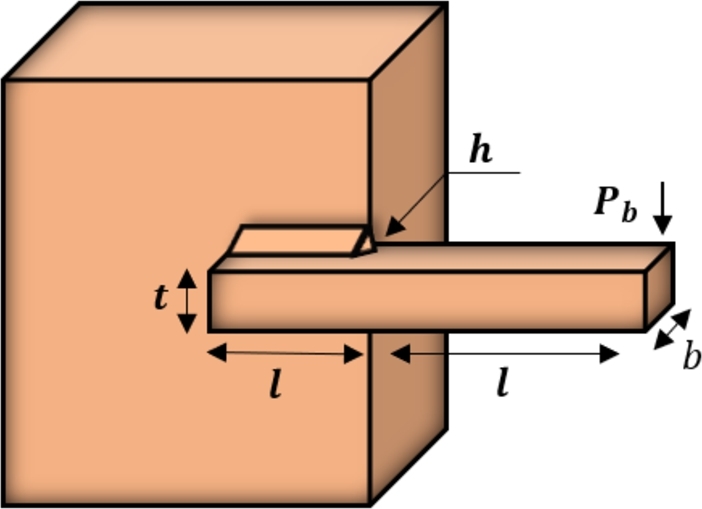
Welded beam design.

The motive of this engineering design is to construct the welded beam by reducing the fabrication cost. The parameters employed for the optimization of the manufacturing costs include the clamped bar's length (*l*), weld thickness (*h*), the thickness of height (*t*) and bar (*b*). The framework and the properties of the welded beam are presented in Fig. 22. Also, with respect to its constraints, as indicated in Eqs. (27) to (33), the minimization function of this problem is mathematically modelled in Eq. (26).

Suppose, $\overset{\to }{w}=[w_{1},w_{2},w_{3},w_{4}]=[h\;l\;t\;b]$

(26) $$Minimizef(\overset{\to }{w})=1.10471w_{1}^{2}w_{2}+0.04811w_{3}w_{4}(14.0+w_{2}),$$

subject to

(27) $$g_{1}(\overset{\to }{w})=\tau (\overset{\to }{w})-\tau_{max}\leq 0,$$

(28) $$g_{2}(\overset{\to }{w})=\sigma (\overset{\to }{w})-\sigma_{max}\leq 0,$$

(29) $$g_{3}(\overset{\to }{w})=\delta (\overset{\to }{w})-\delta_{max}\leq 0,$$

(30) $$g_{4}(\overset{\to }{w})=w_{1}-w_{4}\leq 0,$$

(31) $$g_{5}(\overset{\to }{w})=P-P_{c}(\overset{\to }{w})\leq 0,$$

(32) $$g_{6}(\overset{\to }{w})=0.125-w_{1}\leq 0,$$

(33) $$g_{7}(\overset{\to }{w})=1.10471w_{1}^{2}+0.04811w_{3}w_{4}(14+w_{2})-5\leq 0.$$

Variables range:

$$0.1\leq w_{1}\leq 2,0.1\leq w_{2}\leq 10,0.1\leq w_{3}\leq 10,0.1\leq w_{4}\leq 2,$$

where,

$$\tau (\overset{\to }{w})=\sqrt{{\left(\tau^{\prime }\right)}^{2}+2\tau^{\prime }\tau^{\prime \prime }\frac{w_{2}}{2R}+{\left(\tau^{\prime \prime }\right)}^{2}},\;\tau^{\prime }=\frac{P}{\sqrt{2w_{1}w_{2}}},\;\tau^{\prime \prime }=\frac{MR}{J},M=P\left(L+\frac{w_{2}}{2}\right),\;R=\sqrt{\frac{w_{2}^{2}}{4}+{\left(\frac{w_{1}+w_{3}}{2}\right)}^{2}},\;J=2\sqrt{2w_{1}w_{2}[R^{2}]},\sigma (\overset{\to }{w})=\frac{6PL}{w_{4}w_{3}^{2}},\;\delta (\overset{\to }{w})=\frac{6PL^{3}}{Ew_{3}^{2}w_{4}},P_{c}(\overset{\to }{w})=\frac{4.013E\sqrt{\frac{w_{3}^{2}w_{4}^{6}}{36}}}{L^{2}}\left(1-\frac{w_{3}}{2L}\sqrt{\frac{E}{4G}}\right),P=6000lb,\;L=14in.,\;E=30⁎10^{6}psi.,\;G=12⁎10^{6}psi.,\tau_{max}=13,600psi.,\;\sigma_{max}=30,000psi.,\;\delta_{max}=0.25in.$$

**Figure 23:**
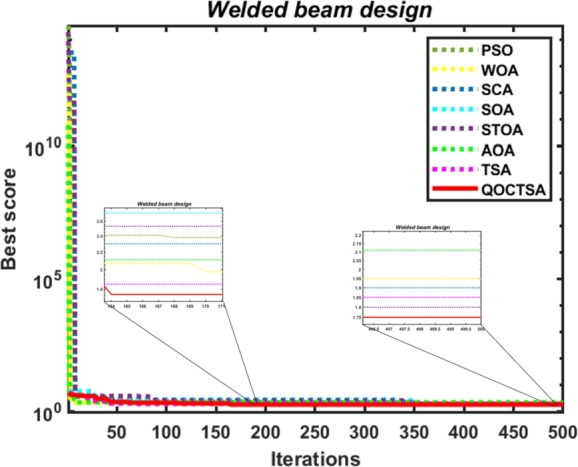
Convergence curve of welded beam design.

Table 20, Table 21 provide the best findings and performance metrics of the QOCTSA with competitive algorithms for the welded beam design problem. From Table 20, it discloses that the QOCTSA has attained the minimum cost of $f(\overset{\to }{(}w)$= 5.870E+03 with the corresponding optimal outcomes of [$w_{1}$, $w_{2}$, $w_{3}$, $w_{4}$]=[2.053E-01, 3.522E+00, 9.154E+00, 2.064E-01] relative to the other optimization algorithms. Table 21 shows that QOCTSA outperforms all performance metrics, including *p*-values, and has the lowest cost, followed by SOA and STOA. Also, from Fig. 23, it can be seen that the enlarged part of the convergence curve of the welded beam is substantially faster and delivers a superior outcome than the standard TSA and other algorithms. Thus, the outcomes of this engineering design exhibit that the proposed QOCTSA has certain benefits over other competing algorithms.

**Table 20:** Best outcomes of the competing algorithms for solving the welded beam design problem.

**Algorithms**	**Optimal values of variables**				**Optimal cost**
	*h*	*l*	*t*	*b*	
**PSO**	2.367E-01	3.118E+00	8.422E+00	2.369E-01	1.836E+00
**WOA**	1.633E-01	4.712E+00	9.001E+00	2.230E-01	1.946E+00
**SCA**	1.945E-01	3.805E+00	9.344E+00	2.153E-01	1.882E+00
**SOA**	1.696E-01	4.489E+00	9.064E+00	2.059E-01	1.803E+00
**STOA**	1.795E-01	4.211E+00	9.038E+00	2.071E-01	1.790E+00
**AOA**	2.009E-01	5.094E+00	1.000E+01	2.014E-01	2.078E+00
**TSA**	2.055E-01	3.152E+00	1.000E+01	2.088E-01	1.870E+00
**QOCTSA**	2.053E-01	3.522E+00	9.154E+00	2.064E-01	**1.750E+00**

**Table 21:** Comparison outcomes of the welded beam design problem.

**Algorithms**	**Best**	**Mean**	**Std**	**Worst**	*p***-values**
**PSO**	1.836E+00	1.836E+00	9.996E-03	1.836E+00	1.319E-06
**WOA**	1.946E+00	2.847E+00	8.822E-01	5.171E+00	1.779E-06
**SCA**	1.882E+00	1.927E+00	6.578E-02	2.073E+00	1.762E-06
**SOA**	1.803E+00	1.815E+00	2.079E-02	1.815E+00	3.273E-01
**STOA**	1.790E+00	1.761E+00	2.294E-02	1.841E+00	8.822E-01
**AOA**	2.078E+00	2.291E+00	2.779E-01	3.031E+00	1.778E-06
**TSA**	1.870E+00	1.844E+00	9.537E-02	2.290E+00	5.884E-06
**QOCTSA**	**1.750E+00**	**1.759E+00**	**8.766E-03**	1.790E+00

### 5.4 Three-bar truss design problem

**Figure 24:**
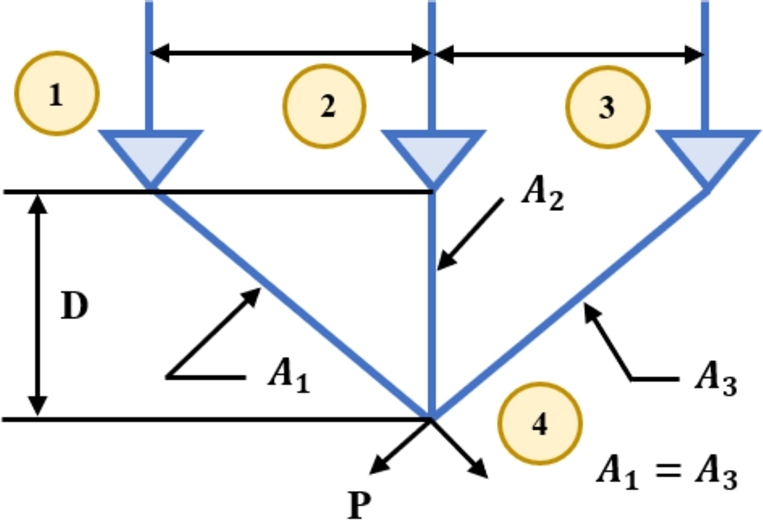
Three-bar truss design.

The key objective of this engineering design is to significantly reduce the truss weight and to determine the most promising outcomes of the two decision variables, such as $A_{1}$ and $A_{2}$. Furthermore, the design of the three-bar truss illustrated in Fig. 24 has number of constraints, including deflection, buckling, and stress. Moreover, the objective function of this design is mathematically expressed in Eq. (34), subjected to the constraints outlined in Eqs. (35) to (37).

Consider, $\overset{\to }{z}=[z_{1},z_{2}]=[A_{1}\;A_{2}]$

(34) $$Minimize\;f(\overset{\to }{z})=(2\sqrt{2}z_{1}+z_{2})\times l$$

subject to

(35) $$g_{1}(\overset{\to }{z})=\frac{\sqrt{2}z_{1}+z_{2}}{\sqrt{2}z_{1}^{2}+2z_{1}z_{2}}P-\sigma \leq 0,$$

(36) $$g_{2}(\overset{\to }{z})=\frac{z_{2}}{\sqrt{2}z_{1}^{2}+2z_{1}z_{2}}P-\sigma \leq 0,$$

(37) $$g_{1}(\overset{\to }{z})=\frac{1}{\sqrt{2}z_{2}+z_{1}}P-\sigma \leq 0,$$

$$l=100cm,\;P=2kN/cm^{3},\;\sigma =2kN/cm^{3},$$

Variable range:

$$0\leq z_{1},z_{2}\leq 1.$$

**Figure 25:**
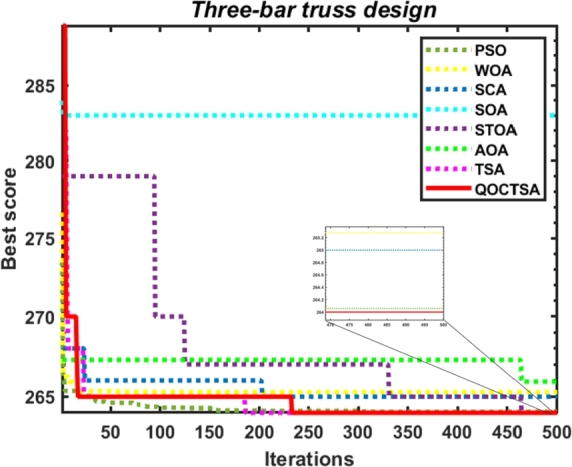
Convergence curve of three-bar truss design.

The performance of the QOCTSA and seven other competing MHAs with several performance metrics are recorded in Table 22, Table 23. Table 22 discloses that the outcomes of QOCTSA have optimized the cost of three-bar truss as $f(\overset{\to }{(}x)$= 6.227E+03 under the optimal solution of [$z_{1}$, $z_{2}$]=[7.936E-01, 3.947E-01] by satisfying the constraints requirements than prior reported algorithms. Furthermore, 23 clearly demonstrates that the proposed approach outperforms all other competing algorithms in terms of performance metrics (‘Best’, ‘Mean’, ‘Std’, ‘Worst’, and ‘*p*-values’). The magnified portion of graphical curve of three-bar truss in Fig. 25 further reveals that the QOCTSA has superior and better convergence accuracy than other algorithms. Thus, the outcomes highlight the superiority and robustness of the proposed QOCTSA in addressing the three-bar truss problem.

**Table 22:** Best outcomes of the competing algorithms for solving the three-bar truss design problem.

**Algorithms**	**Optimal values of variables**		**Optimal cost**
	$A_{1}$	$A_{2}$	
**PSO**	7.887E-01	4.102E-01	2.639E+02
**WOA**	7.440E-01	5.520E-01	2.656E+02
**SCA**	7.688E-01	4.719E-01	2.646E+02
**SOA**	1.000E+00	0.000E+00	2.828E+02
**STOA**	8.005E-01	3.765E-01	2.641E+02
**AOA**	7.899E-01	4.239E-01	2.658E+02
**TSA**	8.008E-01	3.751E-01	2.640E+02
**QOCTSA**	7.936E-01	3.947E-01	**2.639E+02**

**Table 23:** Comparison outcomes of the three-bar truss design problem.

**Algorithms**	**Best**	**Mean**	**Std**	**Worst**	*p***-values**
**PSO**	2.639E+02	2.649E+02	2.033E-01	2.649E+02	NA
**WOA**	2.656E+02	2.652E+02	1.415E+00	2.694E+02	2.369E-04
**SCA**	2.646E+02	2.666E+02	6.477E+00	2.828E+02	NA
**SOA**	2.828E+02	2.703E+02	9.034E+00	2.828E+02	1.953E-03
**STOA**	2.641E+02	2.671E+02	7.142E+00	2.828E+02	NA
**AOA**	2.658E+02	2.662E+02	4.641E+00	2.828E+02	9.409E-05
**TSA**	2.640E+02	2.642E+02	2.138E-01	2.648E+02	NA
**QOCTSA**	**2.639E+02**	**2.641E+02**	**1.322E-01**	2.645E+02

## 6 Conclusion and scope of future work

This paper proposes a novel Quasi-Oppositional Chaotic Tunicate Swarm Algorithm (QOCTSA) to improve the exploration and exploitation abilities of the TSA. This proposed method is developed by the simultaneous integration of the QOBL strategy and the CLS technique with ten distinct chaotic maps. The incorporation of the QOBL technique accelerates convergence and promotes exploration by improving the quality of initial solution sets. In contrast, the implementation of ten distinct chaotic maps in the updating phase of QOCTSA assists in locating optimal values by enhancing the exploitation of the most promising search regions. To evaluate the simplicity and robustness of the proposed algorithm, it has been tested on a diverse set of thirty-three test functions, including the well-known CEC2005 and CEC2019, as well as four real-world engineering design problems, namely tension or compression spring, pressure vessel, welded beam, and three-bar truss design problems. The statistical outcomes demonstrate that the QOCTSA method outperforms the original TSA. Furthermore, when compared to other MHAs, including LSHADE, PSO, WOA, SCA, SOA, and STOA, the proposed QOCTSA method has achieved superior outcomes than the competing algorithms. Therefore, it can be concluded that the QOCTSA method is more effective in tackling engineering design problems than other algorithms. Although QOCTSA performs extensively in solving complex test functions and certain engineering design problems, it does have significant limitations. Despite the manifold benefits mentioned, like other MHAs, it may be prone to getting stuck in local optimal regions if it traverses into an excessive number of singularities or peaks when solving high-dimensional problems, and it may also require more execution time due to the performance of additional loops. Also, the reliability of some of the test functions is not significantly improved while addressing complex multi-modal functions and CEC2019 functions compared to other algorithms. Furthermore, QOCTSA has inadequate performance while endeavouring to solve some challenging real-world applications. Thus, the future study will focus on the following recommendations: (1) The parameters employed in the proposed approach are fixed values obtained through experimentation. Indeed, the algorithm will perform better when the parameters are set to be fixed for various optimization problems. Hence, it will be one of the future works that focus on further enhancing the QOCTSA by dynamically modifying the parameters or incorporating with other successful strategies; (2) the proposed method has significant application potential, including power dispatch problems, feature selection, job scheduling problems, and highly challenging engineering applications, etc.; (3) it can also be expanded to evolve as a multi-objective optimization algorithm or hybridized with other MHAs to improve its efficiency; (4) At last, the outstanding potential of the proposed algorithm suggests that it may be a great choice for solving clustering and binary problems.

## Funding information

No funding was received for conducting this study.

## CRediT authorship contribution statement

**Vanisree Chandran:** Writing – original draft, Methodology, Conceptualization. **Prabhujit Mohapatra:** Writing – review & editing, Supervision, Methodology, Conceptualization.

## Declaration of Competing Interest

The authors declare that they have no known competing financial interests or personal relationships that could have appeared to influence the work reported in this paper.

## Data Availability

Data will be made available on request.
